# A robust platform for high-throughput screening of therapeutic strategies for acute and chronic spinal cord injury

**DOI:** 10.1016/j.isci.2021.102182

**Published:** 2021-02-12

**Authors:** Vaibhav Patil, Enda O'Connell, Leo R. Quinlan, Howard Fearnhead, Siobhan McMahon, Abhay Pandit

**Affiliations:** 1 CÚRAM, SFI Research Centre for Medical Devices, National University of Ireland, Galway, Ireland; 2 Anatomy, National University of Ireland, Galway, Ireland; 3 Genomics and Screening Core Facility, National University of Ireland, Galway, Ireland; 4 Physiology, National University of Ireland, Galway, Ireland; 5 Pharmacology and Therapeutics, National University of Ireland, Galway, Ireland

**Keywords:** Molecular Neuroscience, Cellular Neuroscience, Immunology, Proteomics

## Abstract

Astrocytes and microglia are critical regulators of inflammatory cascade after spinal cord injury (SCI). Existing glial *in vitro* studies do not replicate inflammatory phases associated with SCI. Here, we report an *in vitro* model of mixed glial culture where inflammation is induced by the administration of pro-inflammatory cytokines (tumor necrosis factor-α, interleukin-1β, and interleukin-6) to promote pathologically relevant “acute” and “chronic” inflammatory phases. We observed SCI relevant differential modulation of inflammatory pathways, cytokines, chemokines, and growth factors over 21 days. Mitochondrial dysfunction was associated with a cytokine combination treatment. Highly expressed cytokine induced neutrophil chemoattractant (CINC-3) chemokine was used as a biomarker to establish an enzyme-linked immunosorbent assay-based high-throughput screening (HTS) platform. We screened a 786-compound drug library to demonstrate the efficacy of the HTS platform. The developed model is robust and will facilitate *in vitro* screening of anti-reactive glial therapeutics for the treatment of SCI.

## Highlights

• An *in vitro* MGC model replicates the inflammatory phases associated with SCI
• Differential modulation in NF-κB, MAPK, and immunomodulatory pathways over 21 days
• Change in mitochondrial bioenergetics over seven days
• ELISA-based HTS platform using CINC-3 as a biomarker is established

## Graphical abstract

**Figure:**
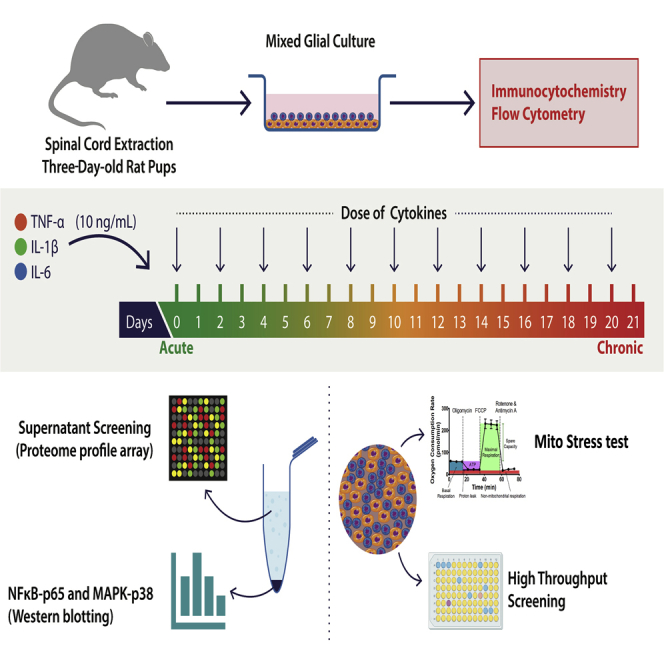

## Introduction

Spinal cord injury (SCI) is a catastrophic event that results in severe primary mechanical trauma followed by more complex secondary injury (Kabu et al., 2015). In the secondary injury process, inflammation is one of the critical hallmarks due to activation of highly motile microglia, astrocytes, and infiltration of blood-borne immune cells at the site of injury (DiSabato et al., 2016). The dual activation (microglia and astrocytes) sparks a burst release of pro-inflammatory cytokines such as tumor necrosis factor-α (TNF-α), interleukin (IL)-1β, and interleukin (IL)-6 at the injury site (Donnelly and Popovich, 2008). These cytokines activate various pathways such as nuclear factor kappa-light-chain-enhancer of activated B cells (NF-κB), mitogen-activated protein kinase (MAPK), nitric oxide (NO) synthase, and chemokine signaling by activating their corresponding receptors which plays a vital role in upregulating the inflammatory cascade (Becher et al., 2017). This leads to the secretion of inflammatory biomarkers such as high mobility group box 1 (HMGB1), NO (Pineau and Lacroix, 2007), and cytokine-induced neutrophil chemoattractant (CINC-3) also known as macrophage inflammatory protein (MIP)-2 (Chio et al., 2019). Glial activation also induces reactive oxygen species (ROS) production, which further elevates the inflammation and upregulates the corresponding biomarkers (Xu et al., 2005). These inflammatory stimuli can persist from acute to chronic phases after the initial trauma (Norenberg et al., 2004). Studying these biomarkers helps us to understand the activation of inflammation and the development of therapeutics in SCI.

Most of the glial *in vitro* studies related to inflammation are focused on studying molecular mechanisms involved in the acute phase and are unspecific with no emphasis on the chronic phase (Alizadeh et al., 2019). Therefore, creating an *in vitro* model that mimics glia-specific chronic inflammatory conditions is paramount. During inflammation, it is known that astrocytes and microglia are involved in cross talk in terms of regulation of inflammation. However, the mechanism of their co-operation is yet to be fully understood (Liu et al., 2011). Also, how they co-ordinate signals with each other under the influence of different cytokines is yet to be fully understood.

Over the last two decades, our understanding of the role of astrocytes and microglia in SCI has led to various strategies to overcome inflammation-induced pathological conditions (Möller and Boddeke, 2016). However, there is a lack of robust *in vitro* models that can provide not only the assessment of the progression of glial cell-derived inflammation but also a method for screening therapeutic interventions which help treatment options for SCI. Although drug screening has been carried out with BV2 microglial cell line (Mutemberezi et al., 2018) and RAW264.7 (Zhang et al., 2019) and THP-1 macrophages (Park, 2017) (Jhang et al., 2015), primary cultures have the advantage of more closely resembling the disease state. Hence, we have introduced a primary mixed glial-based, quantitative high-throughput screening (HTS) platform, using a CINC-3 chemokine, as a marker of inflammation.

Here, we report the development of a cytokine-induced inflamed mixed glial culture (MGC) *in vitro* model to study the acute and chronic inflammatory phases of SCI at the preclinical stage. Studies have demonstrated that astrocytes and microglia respond to the primary mechanical injury and become inflamed as early as day one after SCI (Sun et al., 2019); (Wang et al., 2019); therefore, we have considered one-day postinflammation as “acute” and 21-day postinflammation as “chronic”. The pattern of the expression of NF-κB and MAPK pathways was studied over 21 days following inflammatory cytokine/s and lipopolysaccharide (LPS) treatment. Supernatant profiling was carried out to study the expression of chemokines, cytokines, and growth factors over 21 days. The bioenergetic phenotype of glia and mitochondrial respiration was studied over seven days after treatment. Finally, by using this model, we have demonstrated for the first time that our established model can be used to screen potent anti-inflammatory drugs. As a proof of concept, a CINC-3-based HTS was performed to demonstrate small volume, enzyme-linked immunosorbent assay (ELISA)-based compound primary screening, and further efficacy was assessed using secondary screening.

## Results

### MGC containing microglia and astrocytes showed morphological changes upon cytokine combination treatment

**Figure 1:**
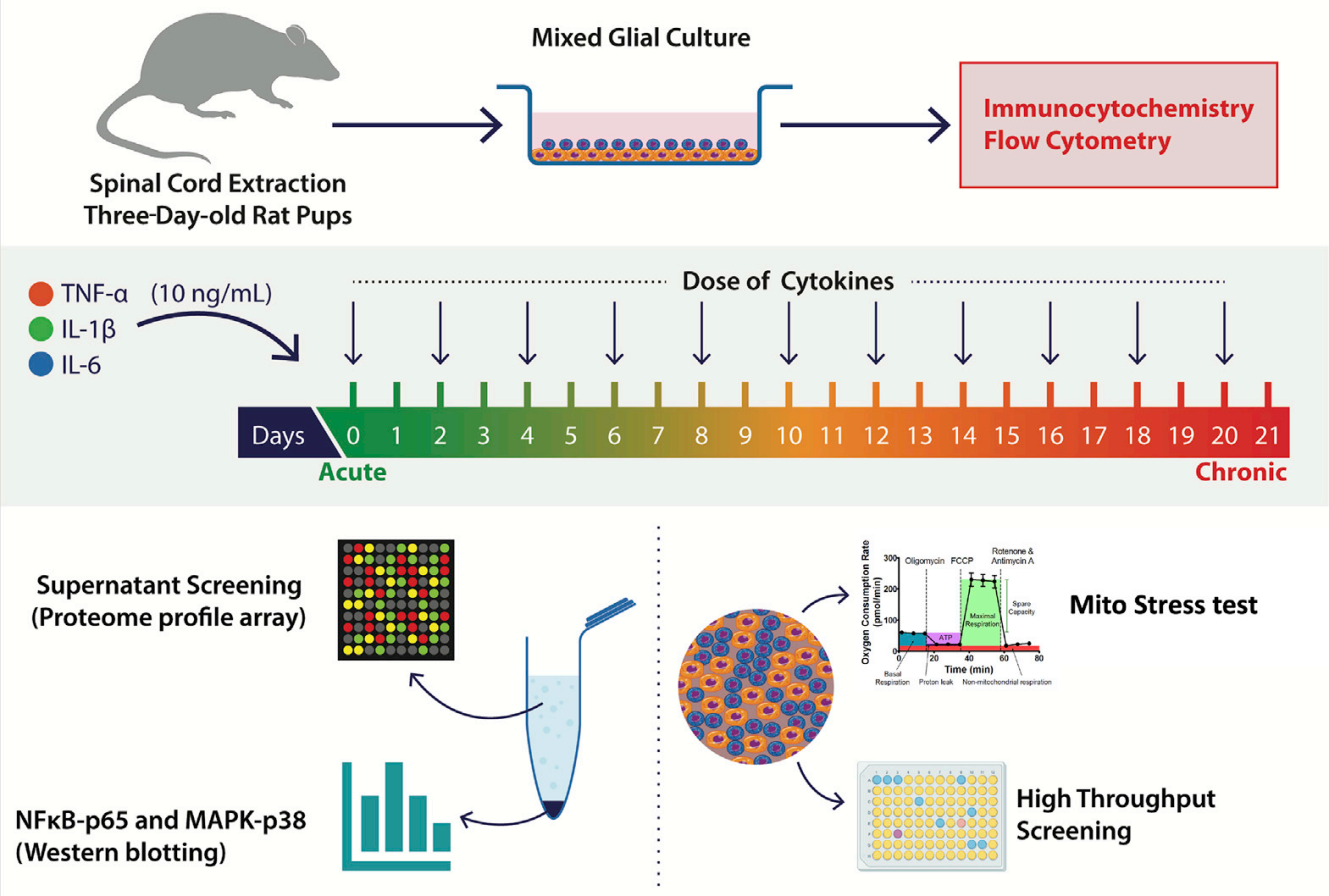
Workflow describing steps involved in the development of the MGC HTS model MGCs were prepared from spinal cords isolated by the hydraulic extrusion technique from three-day-old postnatal rats. Immunocytochemistry and flow cytometry were performed to characterize the MGC, and further quantification of astrocytes, microglia, oligodendrocytes, and neurons was carried out. MGCs were treated with TNF-α, IL-1β, and IL-6 (10 ng/mL per cytokine) in combination/s for one day (acute) to 21 days (chronic). LPS (10 ng/mL) was used as a positive control. Treatments were given every alternate day (n = images from three experimental replicates). The supernatant was assayed using the Proteome Profiler Array (R&D Systems, Inc.). Total protein was extracted, and western blotting was performed to study the activation of NFκB-p65 and MAPK-p38 pathways (n = 3). Mitochondrial function was assessed by using Seahorse Mito Stress Test assay. MGCs were treated with TNF-α, IL-1β, and IL-6 (10 ng/mL per cytokine) for seven days (n = 3). Finally, an HTS platform was established that enabled identification of anti-inflammatory compounds.

**Figure 2:**
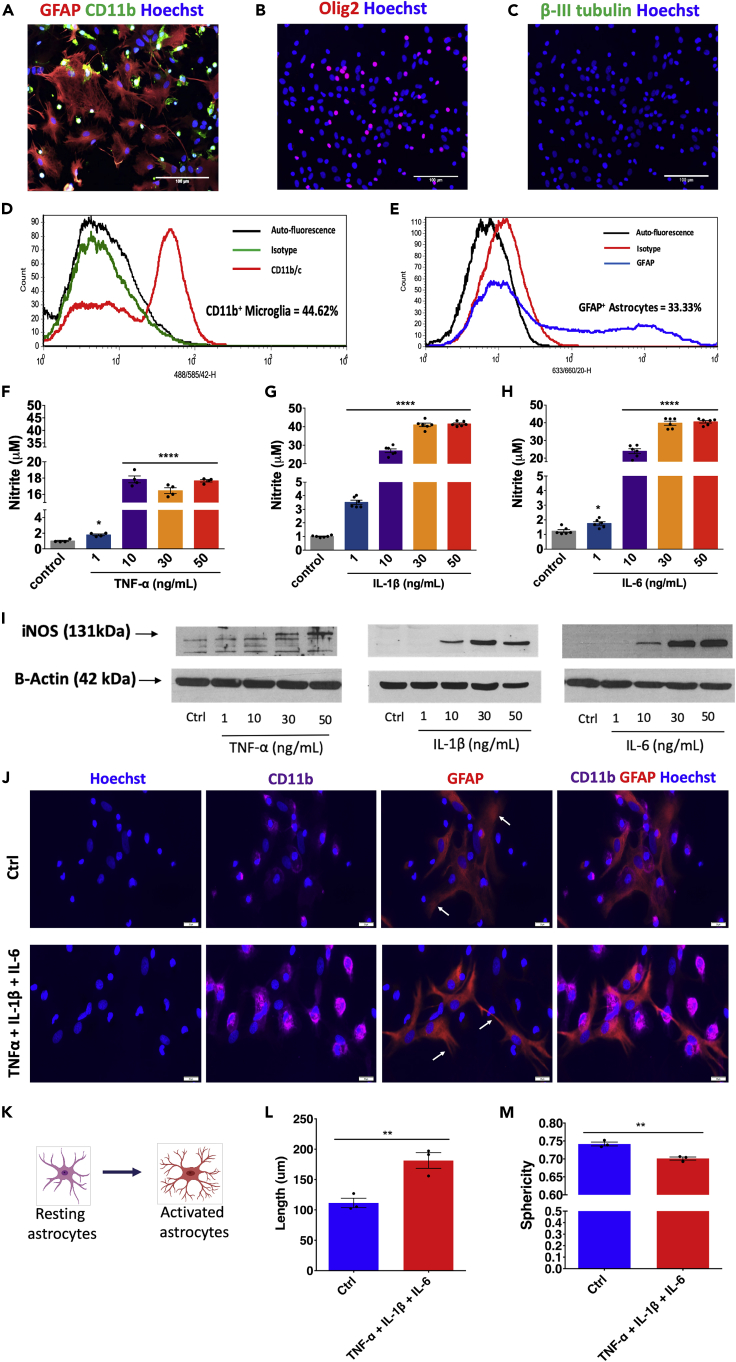
Characterization of MGC and optimization of pro-inflammatory cytokine dose and effect of the treatment on MGC phenotypes (A–C) Immunostaining using GFAP, CD11b, and Olig2 markers showed the presence of astrocyte, microglia, and oligodendrocytes, respectively. The Beta III tubulin staining could not detect any neurons in MGC. n = 3, scale bar = 100 μm. (D–E) Flow cytometry confirms the quantification of astrocytes and microglia in MGC. (F–I) Griess assay and western blotting showing the production of nitrite and iNOS expression, respectively, under different dosage of TNF-α, IL-1β, and IL-6 treatment. A 10 ng/mL dose of each of cytokine was selected to be used for further studies. Data are expressed as mean ± standard error of the mean (SEM), n = two independent experiments each with two-three replicates; ∗∗∗∗p < 0.0001, ∗p < 0.05 compared with the control group (24 hr), one-way analysis of variance (ANOVA), *post hoc* Tukey test. (J) Effect of cytokine combination on astrocytes (GFAP, red) and microglia (CD11b, violet). Scale bar = 20 μm. (K) Change in the morphology of astrocytes from resting to activated phase. Their processes become more ramified upon activation. (L and M) Quantification of astrocytic processes shows processes that were (L) more elongated and (M) showed a decrease in roundness after cytokine combination treatment (24 hr). Data are represented as mean ± SEM, n = 3; ^∗∗^p < 0.01, Mann-Whitney U test (See also Figures S1 and S10).

The study aimed to prepare an *in vitro* model, which will represent the acute and chronic inflammatory state of SCI and use it as an HTS platform to identify anti-inflammatory compounds. The study was divided into various sections to understand immunomodulatory pathways at the translational level, mitochondrial response, and to demonstrate a high-throughput screen for compound screening (Figure 1). Microscopy details demonstrated that the spinal cord-derived MGC population mostly comprised astrocytes (glial fibrillary acidic protein, GFAP^+^) and microglia CD11b^+^ (Figure 2A). These results were further validated by using flow cytometry to confirm the presence of two of glial cell populations; quantification showed the presence of GFAP^+^ astrocytes (33.33%) and CD11b^+^ microglia (44.62%) (Figures 2D and 2E). No B-III tubulin^+^ neurons (Figure 2C) were detected, in line with the absence of any neuronal growth factors or supplements (Gingras et al., 2007) in media. The remaining cells mostly comprised olig2^+^ oligodendrocytes (Figure 2B).

Optimization of cytokine concentrations (TNF-α, IL-1β, and IL-6) involved treating cells with four concentrations of each cytokine (1, 10, 30, and 50 ng/mL) for 24 hr, and the expression of inducible nitric oxide synthase (iNOS) and nitrite release was assessed. All three cytokines (individually) not only changed the morphology of MGC from 10 ng/mL to 50 ng/mL doses (Figure S1A) but also increased nitrite production (Figures 2F–2H) due to the activation of iNOS (Figure 2I). Based on these data, a 10 ng/mL dose of each cytokine was found to be optimal and was used in all further experiments. Moreover, when MGC was treated with the cytokine combination (TNF-α+ IL-1β+ IL-6) with the dose of 10 ng/mL of each cytokine, there were morphological alterations in GFAP+ astrocytic processes (elongated and decreased roundness) (Figures 2J–2M). Microglia (CD11b+) also changed their shape to more circular (ameboid) (Figure S1B). The cytokine combination treatment also increased the nitrite production (Figure S1C). Further, the concentration of LPS sufficient to induce iNOS, nitrite production, and TNF-alpha production (Figures S1D–S2F**)** was determined to be 100 ng/mL**.** LPS was used as a control to compare with the cytokine group and was always added at the same time points on the same number of cells. We have observed that cultures were confluent and alive after 21 days without any treatment. Under cytokine combination treatment, we observed that cells were highly proliferative. In our LPS treatment group, we found that the cells were affected by treatment and that the viability of mixed glia decreased over time (Figure S10).

### Differential activation of NFκB-p65 and MAPK-p38 pathways over 21 days

**Figure 3:**
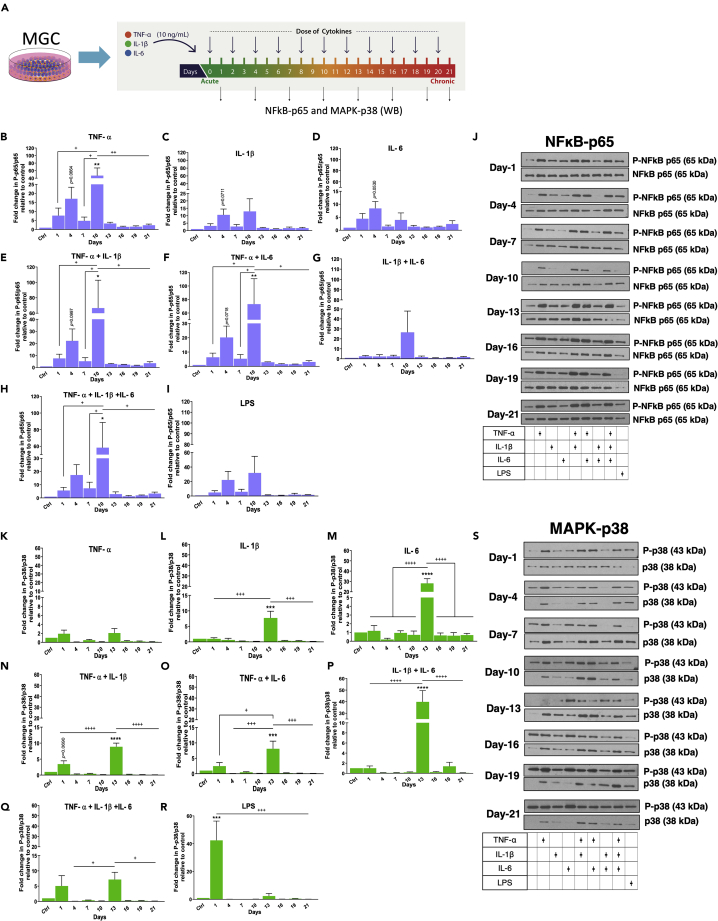
Cytokine combination treatments differentially activate NFκB-p65 and MAP-p38 pathways Western blots show the quantification of the expression of P-NFκB-p65 and P-MAP-p38 from 24 hr up to 21 days upon various combinatorial treatments of TNF-α, IL-1β, and IL-6. The intensity of the P-NFκB-p65 was normalized to NFκB-p65, and the intensity of the P-p38-MAPAK was normalized to p38-MAPAK. (A) Layout of the experiment. (B–H) Upon the treatment of (B) TNF-α, (E) TNF-α and IL-1β, (F) TNF-α and IL-6, and (H) TNF-α, IL-1β, and IL-6 combinations, the pathway was highly activated from the acute to the subacute phase (day one to day ten). However, the combinations of (C) IL-1β, (D) IL-6, and (G) IL-1β and IL-6 treatments did not significantly regulate the pathway over the acute to the subacute phase. (I) LPS did not induce the activation of the pathway significantly. (J) Western blots. All combinatorial treatments did not affect the pathway from day 13 to day 21. (K–Q) (K) TNF-α treatment did not induce significant activation of the pathway from the acute to the chronic phase. Upon treatment of (L) IL-1β, (M) IL-6, (O) TNF-α and IL-6, (P) IL-1β and IL-6, and (Q) TNF-α, IL-1β, and IL-6 combinations, the pathway was significantly activated at day 13. However, (N) TNF-α and IL-1β combination treatment showed the pathway was activated significantly at day one compared to other days except for day 13. In this combination also, the pathway was significantly activated at day 13. (R) LPS induced the activation of the pathway at day one. (S) Western blots. Data are represented as mean ± SEM, n = three experimental replicates ^∗∗∗∗^p < 0.0001, ^∗∗∗^p < 0.001, ^∗∗^p < 0.01, ^∗^p < 0.05 compared with a control group (Ctrl). ^++++^p < 0.0001, ^+++^p < 0.001, ^++^p < 0.01, ^+^p < 0.05; one-way ANOVA, *post hoc* multiple comparison Tukey test (See also Table S1).

It is essential to understand the effect of cytokine alone and in combination on MGC from the acute to the chronic stages of inflammation. Therefore, we selected multiple cytokine combinations and kept LPS as a positive control (Table S1). A dose of 10 ng/mL of each cytokine was given every alternate day to MGC, and on days 1, 4, 7, 10, 13, 16, 19, and 21, media were collected, and proteins were extracted from cells. The extracted protein samples were analyzed by western blotting to detect the expression of NFκB-p65 and p-38MAPK pathways (Figure 3A). This analysis was done to compare time points of each treatment. Analysis of the expression of phosphorylated p65 (for NFκB-p65 pathway) shows each cytokine combination had a variable effect not only on pathway activation but also on the level of activation (Figure 3J). Cytokine combinations with TNF-α alone or in combination with IL-1β and IL-6 and LPS treatment caused phosphorylation of p65 (P-p65) which increased from day one to day four and was downregulated at day seven; however, p65 phosphorylation was not statistically significant (Figures 3B, 3C, 3E, 3F, 3H, and 3I). Moreover, in these cytokine combinations, p65 phosphorylation increased on day 10 and then rapidly decreased from that point until day 21. Although the same trend was observed in the LPS treated group, it was not significant (Figure 3I). In all the groups where TNF-α was involved, strong activation was observed. In contrast, treatments with IL-1β or IL-6 alone did not activate the pathway significantly over 21 days when compared between days (Figures 3C and 3D).

As for phosphorylated p38 (for p38-MAPK pathway), we observed that each cytokine combination also had a variable effect not only on pathway activation but also on a level of activation (Figure 3S). However, unlike the NFκB-p65 pathway, with cytokine combinations and LPS treatments, phosphorylation of p38 did not increase from day one to day four and decreased at day seven (Figure 3S). Moreover, LPS showed a distinct pattern as it significantly activated the pathway at day one (Figure 3R). TNF-α treatment alone did not induce significant activation of the pathway from the acute to the chronic phase (Figure 3K). In IL-1β, IL-6, TNF-α+ IL-6, IL-1β+ IL-6, and TNF-α + IL-1β + IL-6 combinations, the pathway was significantly activated at day 13 and then rapidly decreased from there on till day 21 (Figures 3L, 3M, and 3O–3Q). However, TNF-α+ IL-1β combination treatment showed that the pathway was significantly activated at day one and 13 compared to that on other days (Figure 3N). Overall MAPK-p38 pathway was significantly activated at early time points, and the presence of IL-6 in the treatment group showed higher activation of this pathway at day 13. Therefore, the study of these pathways over a different time course with different cytokine combinations has allowed for a deeper understanding of the progression of the inflammation.

The supernatant (media) from cytokine combination-treated MGC showed differential upregulation of inflammatory molecules and biological functions upon proteome profiling.

**Figure 4:**
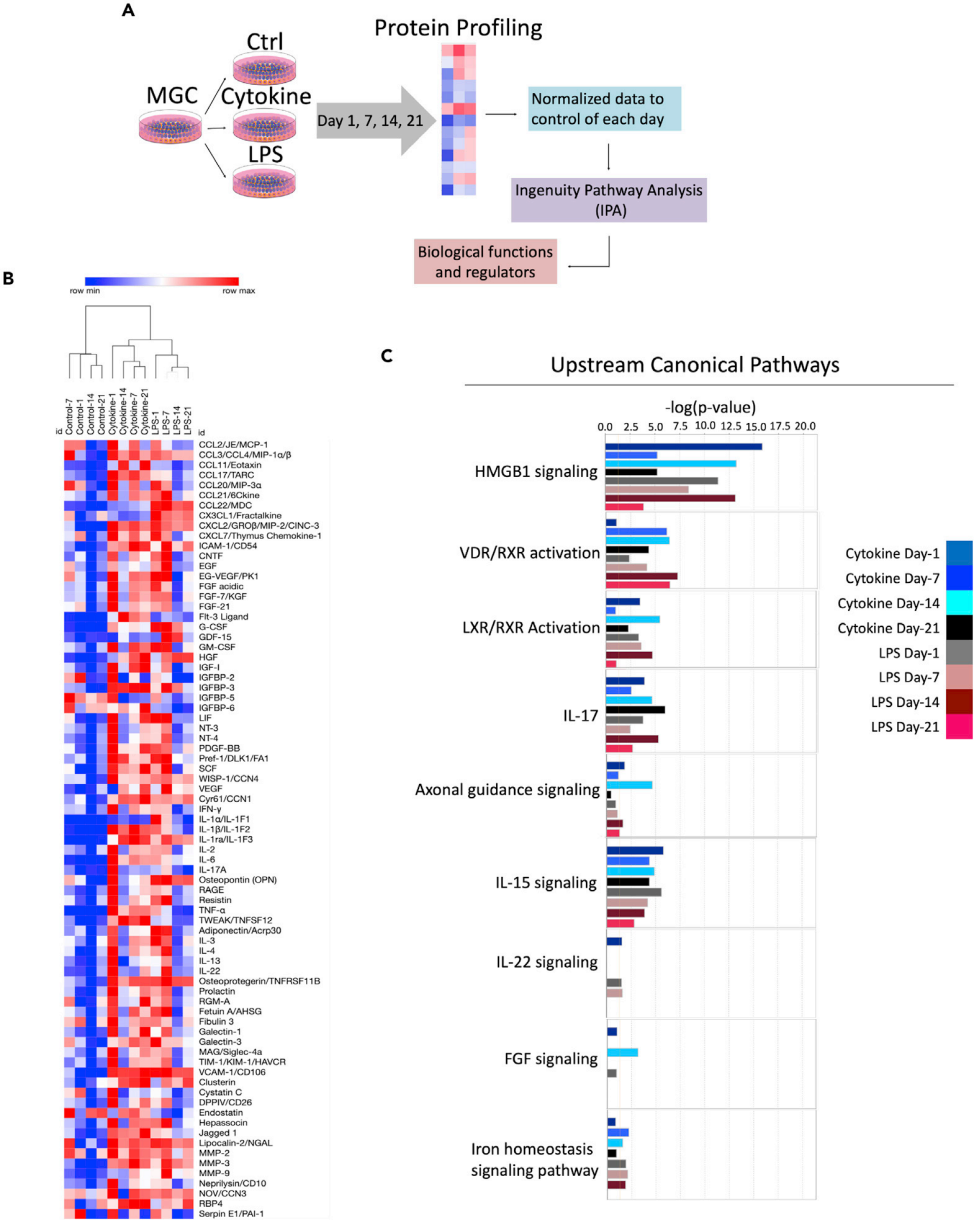
Differential regulation of biological functions and diseases upon cytokine induction (A) Workflow describing steps involved in protein profiling and IPA analysis. (B) Hierarchical clustering analysis of 79 analytes. The mean pixel density analyzed by the Proteome profile array of proteins secreted by MGC in the supernatant. Colors define activation as highly expressed (red) and no expression (blue). Only one cytokine combination (i.e. TNF-α, IL-1β, and IL-6 combination) was used along with LPS as a positive control. Treatment was given from day 1 up to day 21, and at four time points, day 1, day 7, day 14, and day 21, the supernatant was analyzed. The experiment was carried out in three biological replicates, and supernatants were pooled together, and proteome profiler array was performed. Each analyte on the array was printed in duplicate. The values shown per time point are an average of both. (C) The upstream regulators are represented as activation *Z* score. The mean pixel density data obtained from proteome profile array were normalized to control and analyzed in IPA© software with the cutoff of 1.5 for downstream and upstream canonical pathways (See also Figures S2–S5, Data S1 and S2).

The proteome profile array kit was used to assess the expression of chemokines, pro- and anti-inflammatory cytokines, and growth factors secreted by MGC (Figure 4A). Twelve blots showed the various expression patterns of proteins (79 analytes) from day one to day 21 under three conditions (control, cytokine combination, and LPS) (Figure S2). The heat map showed an increase in the production of the number of chemokines (CINC-3, monocyte chemoattractant protein-1, MIP-3α, and chemokine [C-C motif] ligands [CCL] 4), pro-inflammatory cytokines (IL-1ra, TNF-α, IL-1β, IL-6, and osteopontin), growth factors (insulin-like growth factor binding protein [IGFBP]-3, granulocyte colony-stimulating factor, Wnt1-inducible signaling pathway protein-1, and vascular endothelial growth factor [VEGF]), glycoproteins (galectin-3 and vascular cell adhesion molecule [VCAM]-1), matrix metalloproteins (MMP3 and MMP2), neutrophil gelatinase-associated lipocalin, cystatin-C, and cellular communication network factor-3 under the cytokine combination from day one to day 21 (Figures 4B and S4). We also observed a similar trend in the expression of growth factors that were not expressed upon the treatment, except IGFBP-2 and 3 which are involved in the p-53 apoptosis pathway (stress related) (Figure 4B). This is due to the change in fetal bovine serum (FBS) concentration from 10% to 1% in media. We observed upregulation of galectin-3, intercellular adhesion molecule (ICAM)-1, and VCAM-1 in cytokine combination-treated groups. Moreover, using Pearson's correlation hierarchical clustering metric, the treatment groups were separated to several clusters, illustrating the heterogenicity among control, cytokine combination, and LPS treatments. Also, it demonstrated the relationship between the time-dependent treatments (Figure 4B).

Proteome profile data were further analyzed using Ingenuity Pathway analysis (IPA) software to understand the modulation and involvement of upstream canonical pathways, mechanisms, and biological functions related to the regulation of neuroinflammation. We applied the cutoff of 1.5 for downstream and upstream canonical pathways. IPA analysis predicted that the HMBG1 pathway was highly expressed at day one and day 14 upon cytokine combination and day one, seven, and fourteen upon LPS treatment (Figures 4C and S3). This pathway has been well explored in SCI as it stimulates many inflammatory signals. Vitamin D receptor/retinoic X receptor pathway was activated at the later time points (days seven, 14, and 21) in cytokine combination- and LPS-treated groups. Liver X receptor/retinoic X receptor pathway was differentially regulated from day one to day 21, and the pattern of activation was different between the cytokine combination and the LPS groups (Figure 4C). IL-15- and IL-17-mediated pathways showed a similar response between cytokine combination- and LPS-treated groups (Figures 4C and S3). Except for day 14 of the cytokine combination group, the other time points showed unfavorable conditions for axonal guidance signaling. It also predicted that fibroblast growth factor and IL-22 were minimally expressed during all time points emphasizing the presence of inflammatory conditions (Figure 4C). This was further confirmed as we observed upstream regulators plotted as activation *Z* score showed higher expression of pro-inflammatory cytokines (HMGB1, IL-18, IL-17A, IL1, and IL1A) and lower expression of anti-inflammatory cytokines (IL-10, IL22, IL-13, and IL-5). Genes such as suppressor of cytokine signaling (SOCS)-3 and mothers against decapentaplegic homolog-3, which suppresses inflammatory stimulus, were downregulated (Figure S3). Also, phosphatase and tensin homolog (PTEN) reported to be involved in suppression of PI3/AKT/mTOR pathway, also downregulated. There were more ROS and hydrogen peroxide species (Figures S3 and 5); however, the superoxide dismutase-1 responsible for destroying free superoxide radicals was downregulated (Figure S3). This indicates the events causing mitochondrial damage and production of oxygen radicals after inflammation upon SCI. Importantly, these modulations of genes were differentially regulated from day one to day 21 in the cytokine combination groups.

**Figure 5:**
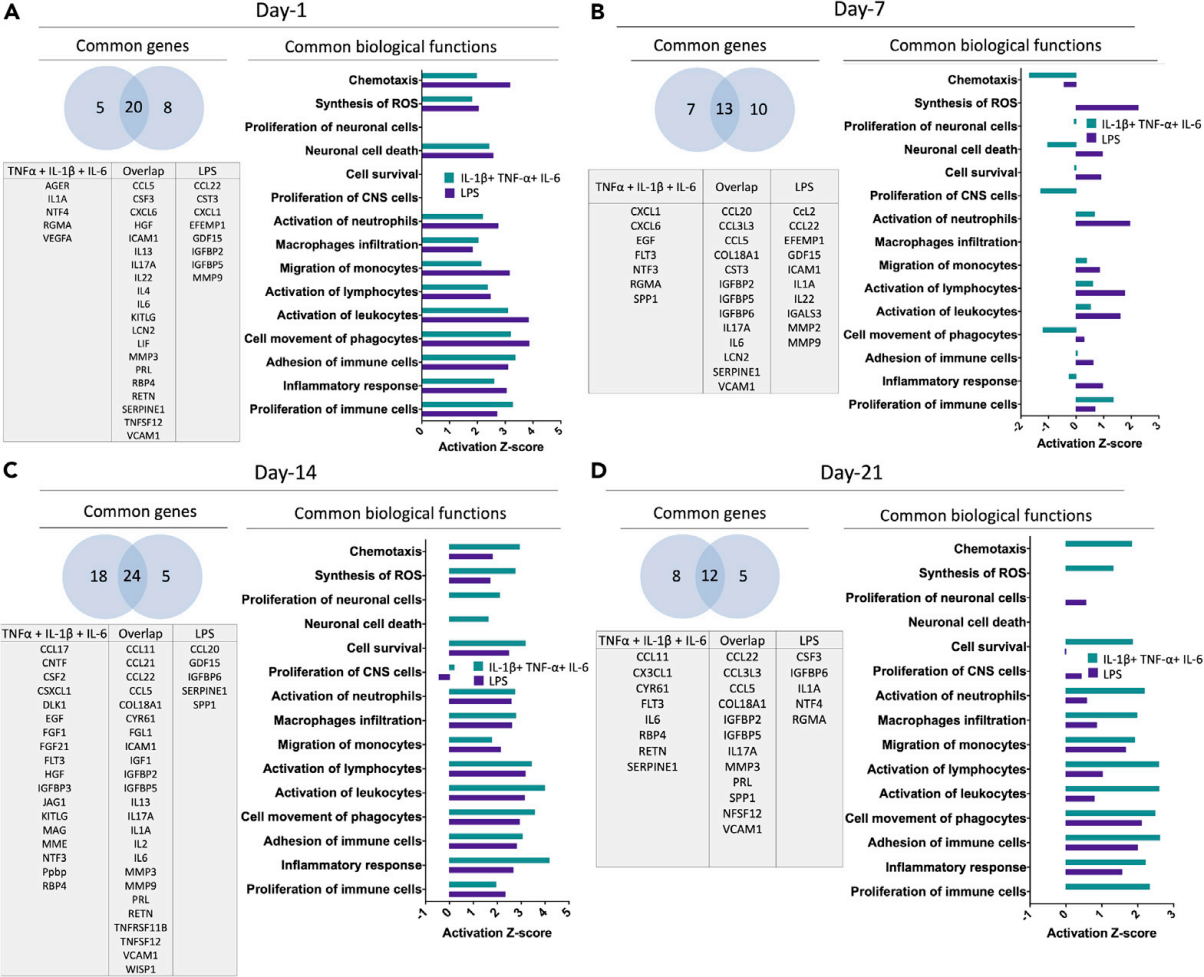
Transcriptome and biological function comparison between cytokine combination and LPS treatment from day one to day 21 (A, B, C, and D) Venn diagram of genes and biological functions analyzed using IPA. Genes commonly expressed between or unique in cytokine combination and LPS treatment are analyzed. Biological functions represented as activation *Z* score show positive values as upregulation, zero as no change, and negative values as downregulation (See also Data S3).

Using IPA, we carried out an analysis to identify essential biological functions associated with cytokine combination and LPS treatments (Figure 5). IPA analysis predicted essential biological functions such as the involvement of immune cells such as macrophages, monocytes, phagocytes, neutrophils, and phagocytes. Cells were highly chemotactic, and their movement was differentially regulated from the acute to the chronic phase (Figure 5). When we investigated common genes expressed between the cytokine combination and the LPS groups, it was evident that these treatments share a 50-75% similarity. However, the number of genes expressed from the acute to the chronic conditions was not constant during individual treatments (i.e. cytokine combination and LPS) nor was their typical expression pattern (Figure 5). Most of these genes were regulated directly or indirectly through NFκB-p65 and MAPK-p38 pathways.

IPA analysis predicated that the genes directly activated by NF-κB complex at day one was VCAM1, IL-6, IL-1α, ICAM1, lipocalin (LCN)2, CCL5, and IL-4 whereas MMP3, advanced glycosylation end-product specific receptor, IL22, IL-13, CXCL6, and IL-17A were indirectly activated. Also, VEGF and SERPINE1 were inhibited (Figure S4A). Most of the genes activated were responsible for eliciting an inflammatory response. In contrast, at day 21, VCAM1, IL-6, and CCL5 were directly activated by the NF-κB complex and MMP3, CCL22, CCL11, IL-17A, and C-C motif chemokine ligand 3-like 3 were indirectly activated which were also responsible for the inflammatory response. Moreover, IGFBP2, SERPINE1, collagen type XVIII alpha 1 chain, and CX3CL1 were downregulated (Figure S4B). Compared to NFκB-p65 pathway, MAPK-p38 pathway had different degrees of response as few genes (VCAM1, IL-1α, ICAM1, MMP3, CCL5, IL-6, CSF3, IL-17A) were activated and in an indirect way on day one and day 21 (Figure S5).

### Mitochondrial dysfunction and higher ROS production in MGC after cytokine combination treatment

**Figure 6:**
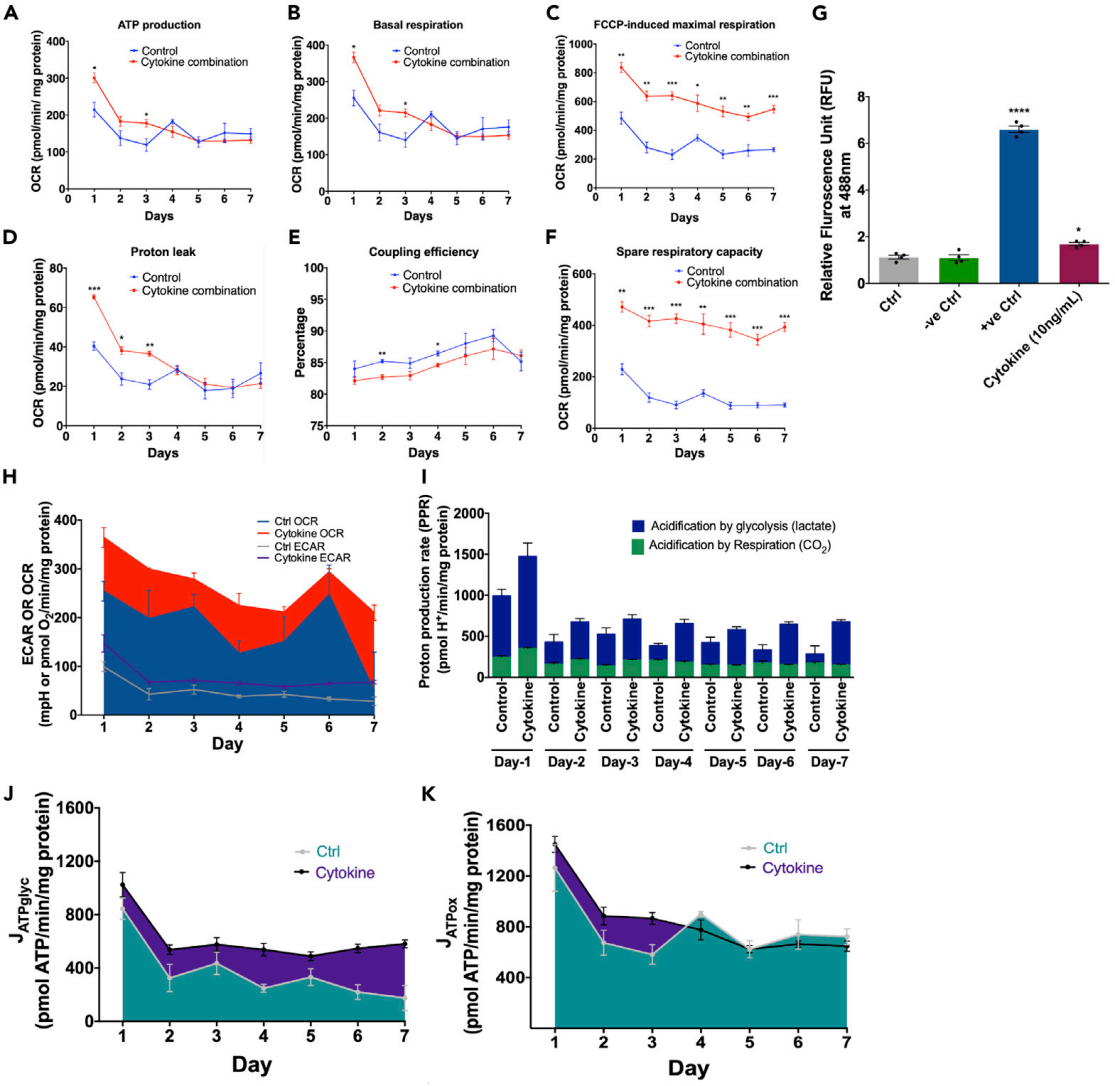
A cytokine combination treatment increases OCR, respiration, ATP production, and proton leak and decreases coupling efficiency and membrane potential over seven days (A–F) All parameters were calculated as a function of a cytokine combination treatment. For this, total protein per well was calculated using a BCA protein quantification assay, and data were normalized against it. Data are represented as mean ± SEM, n = three independent experiments. ∗p < 0.05, ∗∗p < 0.01, ∗∗∗p < 0.001 vs respective day of control. A student t-test was performed to test the difference between the treatment and the respective day of control. (G) ROS species detected using an ROS-superoxide assay confirms an increase in the oxidative stress in MGC. Negative control (-ve Ctrl): ROS inhibitor (N-acetyl-L-cysteine), positive control (+ve Ctrl): ROS inducer (Pyocyanin). Data are represented as mean ± SEM, n = four experimental replicates, ∗p < 0.05, ∗∗∗∗p < 0.001 compared with the Ctrl (control) group. (H) Basal ECAR and OCR plotted on the same axis obtained from control and cytokine combination treatment to MGC. (I) Rate of extracellular acidification caused by glycolysis by lactate production and respiration by CO_2_ production from day one to day seven. Both control and cytokine combination treatment groups showed an increase in acidification by glycolysis rather than by respiration, whereas compared to the control, the cytokine treatment increases glycolysis-based acidification. However, there was no difference between the control and the cytokine treatment group in acidification by respiration. (J) Data from basal ECAR and OCR have been converted to the rate of ATP production by glycolysis using formula. (K) Data from basal ECAR and OCR have been converted to the rate of ATP production by oxidation using the formula. Data are represented as mean ± SEM, n = three experimental replicates (See also Figures S6 and S9 and Data S4).

Seahorse Mito Stress assay was performed to assess mitochondrial function in MGC upon cytokine stimulation. Oxygen consumption rate (OCR) (Figures S6B, S6E, S6H, S6K, S6N, S6Q, and S6T) and extracellular acidification rate (ECAR) (Figures S6C, S6F, S6I, S6L, S6O, S6R, and S6U) were measured with the sequential addition of oligomycin (1 uM), FCCP (2 uM), and rotenone/antimycin (0.5 uM). We observed an increase in the OCR and ECAR in the cytokine treated group, which was expected as pro-inflammatory cytokines activate glial cells (Figures 6H). The cytokine group showed more acidification by glycolysis than the control group at each day of treatment (Figure 6I).

Mitochondrial activity-defining parameters were determined using OCR. The cytokine treated group showed higher maximal respiration (Figure 6C) and spare respiratory capacity (Figure 6F). The basal respiration (Figure 6B), ATP production by oxidation (Figure 6A), and its rate of production (Figure 6K) were higher in the cytokine treated group from day one to day three, but from day four to day seven, there was no difference compared to the control group. In contrast, the rate of ATP production by glycolysis was higher in the cytokine-treated groups at all time points (Figure 6J). These data correlated with the proton leak parameter, which determines whether there is mitochondrial membrane depolarization (Cheng et al., 2017). This parameter is often considered a hallmark of mitochondrial damage, which was further confirmed by calculating coupling efficiency (an efficiency to couple proton translocation across the mitochondrial membrane to ATP production), as it was lower in the cytokine-treated group (Figure 6D). In addition, cytokine combination treatment altered the ATP-induced endogenous intracellular Ca^2+^ signaling (Figure S9). Further, we used an ROS-superoxide detection kit to detect hydrogen peroxide, peroxynitrite, hydroxyl radicals, NO, and peroxy radical production in cells and found that ROS was significantly increased in the cytokine-treated MGC (Figure 6G).

### High-throughput drug screening paradigm

Our proteome profile array has shown upregulation of different chemokines, and after careful analysis, we identified that among all chemokines, CINC-3 was found to be highly expressed in the cytokine treatment (Figures S7 and 4C) and CINC-3 expression was thus used as a biomarker for drug screening. Upon cytokine stimulation, we found significantly high secretion of CINC-3 from glial cells from six hr to 48 hr (Figures S8A–S8F). The Z′ factor calculated to identify the assay suitability was found to be 0.96 (very high) between the control and the cytokine-treated group at 24 hr (Figure S8G). Therefore, we decided to use 24 hr as the biological time point to assess the anti-inflammatory effect of drugs.

**Figure 7:**
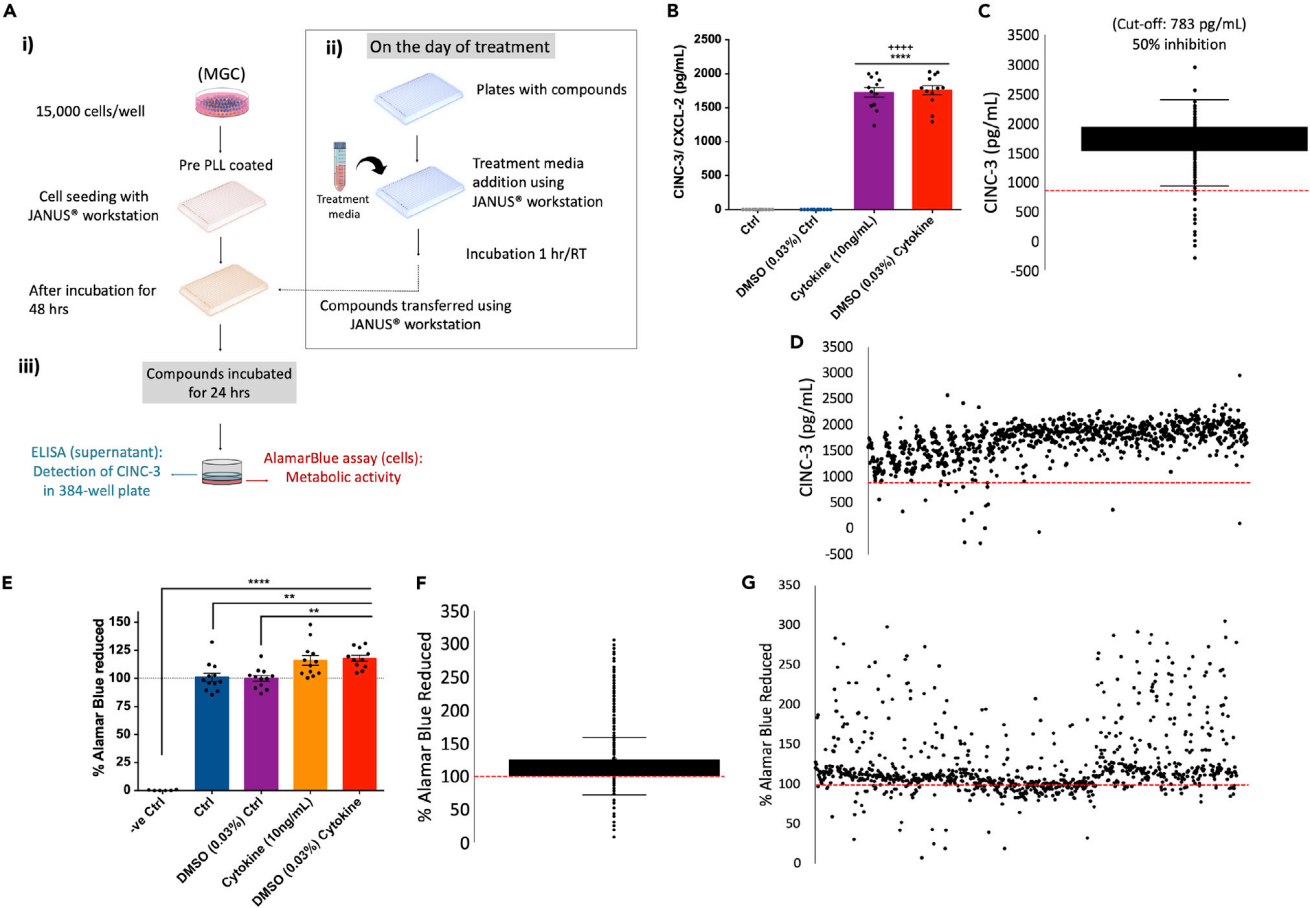
HTS optimization, validation, and its use to identify novel targets for inflammation (A) Workflow for high-throughput screening. (i)15,000 cells/well/50 uL were seeded in pre-PLL-coated 384-well plates and were left to grow for two days. (ii) After two days, 50 uL of the treatment media (without FBS and with cytokine combination) was added into compound plates and incubated for an hour. The treatment media containing compounds was transferred to cell plates after removal of original media. (iii) Compounds were incubated with cells for 24 hr. Further, ELISA was performed on the supernatant to assess CINC-3 expression from each well and alamarBlue assay on cells. All the steps were carried out using a JANUS workstation. There were three compound plates, and each plate contained four replicates of controls (Ctrl, DMSO (0.03%), cytokine combination (IL-1β+ TNF-α+ IL-6, 10 ng/mL each), and DMSO + cytokine combination. (B) Increase in the CINC-3 expression after cytokine combination treatment. Data are represented as mean ± SEM, n = 12, ^∗∗∗∗^p < 0.0001 compared with Ctrl, ^++++^p < 0.0001 compared with DMSO (0.03%) Ctrl; one-way ANOVA followed by *post hoc* Tukey test. (C) Three plates pulled assay points (box plot). Red dotted line: cutoff point (i.e. 783 pg/mL) for 50% inhibition of CINC-3. (D) Three plates pulled assay points (scattered dot plot). (E) alamarBlue assay showing metabolic activity after cytokine combination treatment. Red dotted line: Normalized and compared with the control (i.e. 100%). Data are represented as mean ± SEM, n = three experimental replicates with four technical replicates, ^∗∗∗∗^p < 0.0001, ^∗∗^p < 0.01; one-way ANOVA followed by *post hoc* Tukey multiple comparison test. (F) All assay points (box plot). (G) All assay points (scattered dot plot) (See also Figures S7 and S8 and Table S2).

We repeated the 24 hr time point for this assay using JANUS workstation and multidrop. We obtained similar results with Z′ factor of 0.79 between the control and the cytokine-treated group (Figure 7B). As described in Figure 7A, this screen was carried out in four days on a 786-compound library (Enzo Life Sciences) to screen anti-inflammatory drugs. MGC cells were seeded in plates two days *in vitro*, and then, the cytokine treatment and drug administration to cells were carried out. On the final day, CINC-3 detection and alamarBlue assay were performed. The data were analyzed, and a cutoff of 50% inhibition for CINC-3 production (i.e.783 pg/mL) was applied to identify anti-inflammatory compounds (Figures 7C and 7D). The alamarBlue assay performed after ELISA showed that there was no depletion in metabolic activity upon cytokine treatment (Figure 7E) and more than 95% of drugs had no harmful effect on metabolic activity (Figures 7F and 7G). Based on the cutoff value, we obtained a primary list of the drugs (Table S2). This list contains cytotoxic, corticosteroids, anti-bacterial, and anti-fungal compounds.

**Figure 8:**
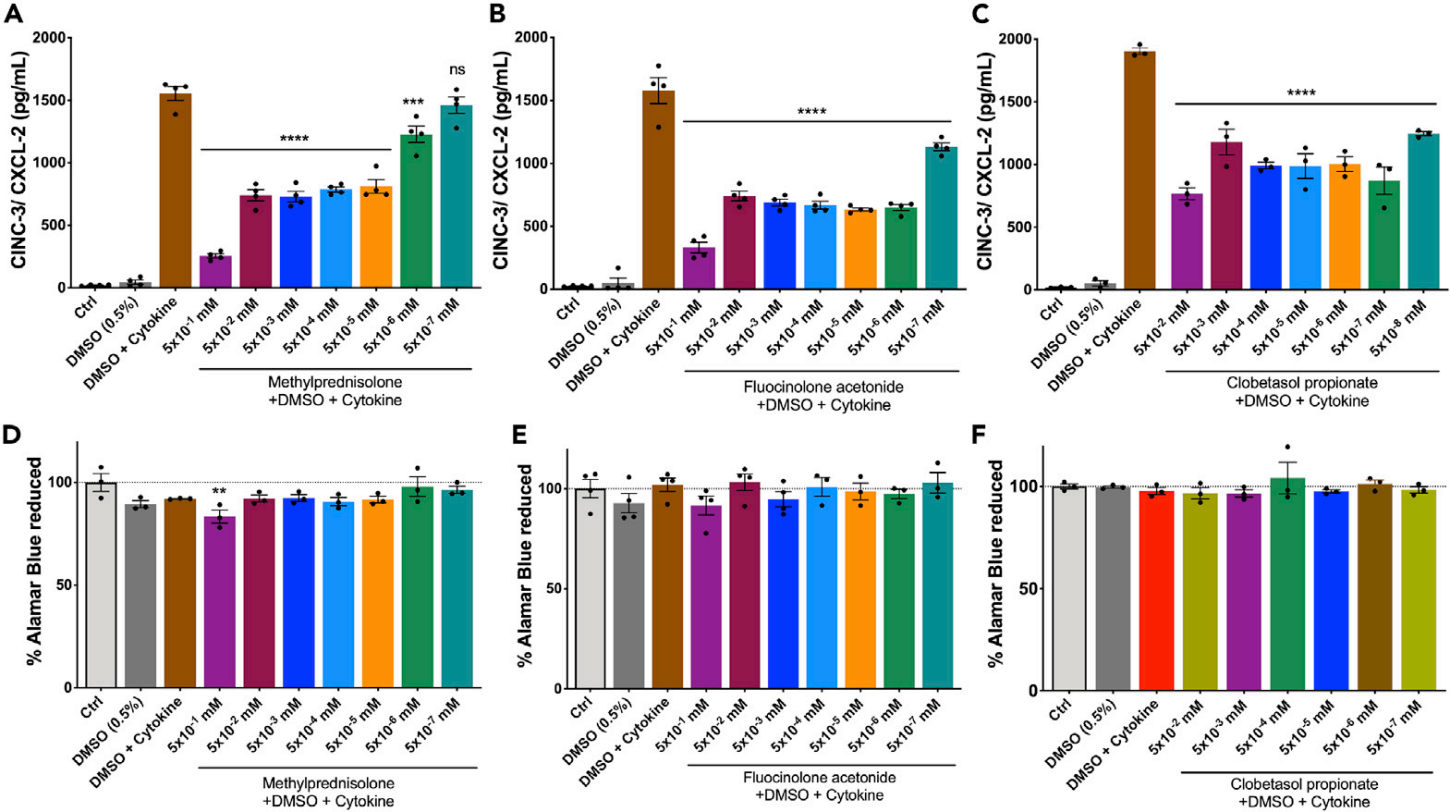
Validation of HTS—dose-dependent significant reduction of CINC-3 production upon corticosteroids treatment during secondary screening (A) Methylprednisolone treatment. (B) Fluocinolone acetonide. (C) Clobetasol propionate treatment. (D–F) There were no metabolic changes upon drug induction. Data are represented as mean ± SEM, n = three-four experimental replicates. For (A-C), ∗∗∗∗p < 0.0001, ∗∗∗p < 0.001 vs DMSO + cytokine group; for (D-F), ∗∗p < 0.01 vs Ctrl group. One-way ANOVA followed by post hoc Tukey test (See also Figure S11 and Table S2).

We performed dose-response studies to validate the HTS platform. We assessed ELISA (CINC-3) on the supernatant collected from wells and alamarBlue assay on cells to assess metabolic activity. As expected, we showed that three of the corticosteroids, methylprednisolone, fluocinolone acetonide, and clobetasol propionate, reduced CINC-3 expression in a dose-dependent manner (Figures 8A–8C) without affecting the metabolic activity of MGC (Figures 8D–8F). Very high doses of these drugs caused cell death due to drug precipitation, as they were water insoluble (data not shown). In addition, we also confirmed the GFAP protein expression in astrocytes (in MGC) upon drug treatments along with cytokine combination. The protein levels were unaltered under these treatments (Figure S11).

## Discussion

The inflammation associated with SCI occurs due to activation of microglia and astrocytes (Popovich et al., 1997); this is followed by glial scar formation because of reactive astrogliosis (activation of astrocytes). These are the significant events which are responsible for the death of healthy oligodendrocytes and neurons around the lesion site, which sometimes leads to irreversible damage (Fitch and Silver, 2008). In the field, there are no appropriate well-characterized and robust *in vitro* models to study the cumulative response of astrocytes and microglia in different stages of inflammation. Here, we have established an MGC prepared from the spinal cord of postnatal rats. To the best of our knowledge, this is the first study of an *in vitro* model, which represents the acute and the chronic inflammatory state for SCI. Unlike single-cell-type screening models, in which separation of microglia and astrocytes pose another level of complexity in the procedure, this method provides a standardized and an easy way of culturing the glia and using the setup as a screening platform.

MGCs are being prepared from spinal cords, either embryonic or postnatal. Both tissue types show variability in terms of the type of cells and the percentage of them (Malon and Cao, 2016) (Beaudet et al., 2015). Here, we used postnatal three-day-old rats to culture spinal cord MGCs. The culture mostly comprises GFAP+ astrocytes and CD11b+ microglia with a small fraction of olig2+ oligodendrocytes. Due to the deliberate lack of specific growth factors and supplements in the medium, the culture was devoid of β-III tubulin + neurons. This allowed the culture to focus on only glia. The choice of using MGC instead of astrocytes or microglia alone is due to the complexity of the inflammatory process. There are reports where microglia-mediated inflammatory responses activate astrocytes via P2Y1 receptors during brain trauma (Shinozaki et al., 2017). Also, reactive microglia activate A1-type reactive astrocytes (Liddelow et al., 2017). Therefore, understanding inflammation and proposing a suitable model for a high-throughput platform needs both cell types, which was missing in most of the earlier available models (Foresti et al., 2013). The shape of astrocytes and microglia is well known to be one of the features of their activation. Astrocytes become hypertrophied as processes stained with GFAP become elongated and thicken upon activation (Cekanaviciute and Buckwalter, 2016). Previously, it has been shown that under LPS stimulation, microglial morphology changes as they become more ameboid (M1 type) in shape upon activation (Tam and Ma, 2014). These changes were similarly observed in our MGC model upon cytokine combination treatment. Based on these observations, we believe that for these characteristic shape changes, it is necessary that both glial cell types be present in direct contact with each other. Microglia response to an initial insult to the injury causes activation and chronic inflammatory stimulation leads to priming of microglia. This phenomenon is observed upon traumatic brain injury and SCI (Norden et al., 2015). Our MGC cultures comprise 45% of microglia. The primed microglia become more proliferative as we have seen in our study. Microglial priming leads to the production of pro-inflammatory cytokines and chemokines, which we have noted in our proteome profiling data. We have seen an increase in the number of microglia in culture upon cytokine combination treatment. There have been studies where microglial activation is caused by one or more of the pro-inflammatory cytokines such as TNF-alpha, IL-1beta, or IL-6. We have used a media control as a negative control and LPS as a positive control. These control groups were used as a baseline for glial activation/priming. Our studies corroborate the recent studies that have shown that primed microglia activate reactive astrocytes (Liddelow et al., 2017) (which are present in our MGC model).

One of the significant challenges in studying inflammation in the spinal cord context is understanding its acute and chronic phases. To the best of our knowledge, there are currently no models which are available to assess the behavior of astrocytes and microglia in the acute and the chronic phases. To address this mechanism, we treated MGC for 21 days, and a specific cytokine dose was given on alternate days. The choice of the alternate day was to give half a fresh media with the treatment combination. It will allow cells to have an energy source and mimic the environment where pro-inflammatory cytokines are present at the injury site. For 21 days, the impact of each pro-inflammatory cytokine on the NF-κB and MAPK pathways was studied as they are involved in the modulation of inflammation (Santa-Cecília et al., 2016) and proliferation and differentiation (Stipursky et al., 2012) of glial cells. More importantly, the levels of TNF-α, IL-1β, and IL-6 increased and peaked at an early stage (earlier than 12 hr) after SCI and remained detectable up to 120 days (Rodríguez-Barrera et al., 2020)*.* TNF-α is known for its pleiotropic effects and is considered one of the strong mediators of the NFκB-p65 pathway (Hayden and Ghosh, 2014). Therefore, in the combinatorial cytokine treatments, the presence of TNF-α showed higher activation of this pathway at time points from day one to day ten. Its activation has a strong effect on chronic modulation of astrocytes as seen in astrocyte culture models in amyotrophic lateral sclerosis (Crosio et al., 2011). Chemokines regulate inflammatory processes through a chemokine signaling pathway that is influenced through the activation of NF-κB pathway (Richmond, 2002). We did not see the production of any anti-inflammatory cytokines or growth factors in the cytokine-treated groups, which confirms that only pro-inflammatory pathways were upregulated. Generally, growth factors are involved in regeneration processes, and during inflammation, their expression level decreases (Anderson et al., 2018). Moreover, we observed upregulation of galectin-3, ICAM-1, and VCAM-1 (glycoproteins) in treated groups which are known to participate in the inflammation process (Rabinovich and Toscano, 2009). We observed a second peak of the activation of NFκB-p65 and p38-MAPK pathway at day 10 and 13, respectively, which is a sign of reactivation of microglia. There is evidence that in SCI, during inflammatory phase, microglia adopt a mixture of M1 (classical activation) and M2 (alternate activation). It has been reported that after primary activation of microglia in the first three to seven days after injury, they reactivated again at around 14 days after injury (Akhmetzyanova et al., 2019) (Bellver-Landete et al., 2019) (Kigerl et al., 2009). Moreover, continuous inflammatory stimulation can contribute to a second phasic immune response, with the predominance of the M1 phenotype (Akhmetzyanova et al., 2019). As mentioned before, microglial activation leads to changes in astrocyte phenotypes/functions and with the second phasic response can lead to further astrocyte activation causing higher activation of them. As explained above, the regulations of these transformations of glia are controlled by canonical pathways including NF-κB and MAPK pathways. In our proteome profiling, we confirm these findings as we observed the second wave of activation of inflammatory pathway modulators at day 14, which correlates with the timeline.

The IPA regulator network analysis predicated few biological functions such as the involvement of macrophages, monocytes, phagocytes, neutrophils, and phagocytes. This can be correlated with the SCI *in vivo* data, which reported that after the injury, these immune cells reach the site of injury and play a critical role in the activation of glial cells (Trivedi et al., 2006). As microglia are resident macrophages of the central nervous system, many molecules secreted by activated microglia may have a similarity with the secretome of immune cell types. It can also be predicted that from day one and day seven, the cytokine-treated group had a favorable condition for glial proliferation, which also support the findings of higher OCR, ECAR, and glycolysis upon cytokine induction. We observed that the NF-κB and MAPK pathway differentially regulated chemokines, cytokines, growth factors, MMPs, and glycoproteins under cytokine treatment from the acute to the chronic phase of inflammation. This was further confirmed as our IPA regulator network analysis also predicted the same pattern of activation.

Moreover, the molecules controlled by this pathway were also responsible for the induction of an inflammatory response. Day one showed high activation of the NF-κB complex and higher neuroinflammatory response, whereas on day 21, this was decreased. This pathway had either a direct or indirect effect of critical regulators responsible for inflammation, whereas MAPK pathway indirectly regulated neuroinflammatory response at day one and day 21 by regulating few genes. Changes in the expression of proteins had a significant effect on several inflammatory conditions which mimic modulations observed during different stages of SCI. This includes chemotaxis, death of healthy neurons, and infiltration of macrophages, monocytes, leukocytes, and lymphocytes, and synthesis of ROS. Surprisingly, we observed that even though cytokine combination treatments were given every alternate day, none of them kept the pathway activated for a long time.

Upon analyzing upstream regulators, we found PTEN was downregulated. It is a negative regulator of PI3/AKT/mTOR pathway (Chen et al., 2016). Activation of PI3/AKT/mTOR pathway is necessary in glial scar formation (Luan et al., 2017). PTEN has several downstream effects such as cellular growth cycle tuning and limiting uncontrolled cell growth (Rafalski and Brunet, 2011). In our experimental setup, we observed a higher metabolic rate and associated higher proliferation of glia, particularly microglia, after the cytokine combination treatment. This supports our finding of upregulation of mTOR and downregulation of PTEN. Also, suppressor of cytokine signaling 3 which negatively controls the cytokine mediated inflammation (Okada et al., 2006) is also downregulated.

We also assessed the functionality of mitochondrial activity during inflammation. It has been well reported that under inflammation stimuli (Joshi et al., 2019) or several pathological conditions (Hou et al., 2019), mitochondrial changes will have severe impacts on alleviating the disease conditions. The OCR is the direct measurement of mitochondrial electron transport rate, and ECAR is proportional to the metabolic activity of the cells. Therefore, an increase in the ECAR can be correlated with higher metabolic activity. This is typically measured by detecting lactate production. At neutral pH, when glucose is converted to lactate, it releases protons during glycolysis making medium acidic by the formation of carboxylate anion. Measurement of this provides a direct and quantitative glycolytic rate. During cytokine combination treatment, astrocytes and microglia become reactive, especially microglial rate of proliferation, which increases drastically. This subsequently increases glycolysis as there is a high demand for ATP, causing increased mitochondrial activity and an increased OCR. In this study, we observed that a cytokine combination treatment caused an increase in oxidative phosphorylation and ATP production from day one to day three. Mitochondrial basal respiration and linked ATP production also reduced after the fourth day until day seven. During cytokine combination treatment, glial metabolism increases and so does the glucose consumption. It is well known that immune cells with quiescent and anti-inflammatory phenotype primarily rely on fatty acid metabolism, whereas pro-inflammatory phenotypic immune cells use glycolysis as the main source of ATP production (Nonnenmacher and Hiller, 2018). This increases lactate production via glycolysis and CO_2_ production via mitochondrial oxidative phosphorylation (respiration) which acidifies the medium. Using OCR and ECAR values, we calculated the proton production rate and found that acidification by the glycolysis process was higher than acidification by respiration. Inflammation triggered in MGC caused mitochondrial respiration impairment as proton leak was increased and coupling efficiency was decreased from day one to day seven. This was caused by a greater number of cells losing mitochondrial membrane potential upon cytokine combination treatment. Due to the imbalance between energy need and the capacity of mitochondria, there is leakage of protons from the inner membrane of mitochondria. In the end, this leads to the production of ROS (Visavadiya et al., 2016) which can persist for weeks to months after SCI (Donnelly and Popovich, 2008). According our observation to IPA prediction showed ROS was prevalent during chronic condition upon cytokine induction in MGC.

In proteome profiling, one of the chemokines, CINC-3, which belongs to the family of CXC chemokine (Shibata et al., 2000) was found to be increased after cytokine treatment. It plays the role of chemoattractants in immune responses. CINC-3 has been well studied in rat models (Takano and Nakagawa, 2001) in inflammatory conditions. CINC-3 has a role in neutrophil requirement at the site of injury; however, it has never been used to test inflammation in MGC. Also, it has never been used as a biomarker for drug screening. Therefore, we decided to further validate the production of CINC-3 during cytokine treatment and found that there was a significant increase after the treatment. We confirmed that this assay is with huge potential as Z′ factor value was close to ‘1’ and our assay reflects in the category of ‘Ideal assay’. We further successfully demonstrated the low volume 384-well plate ELISA method-based detection of CINC-3. Here, we used only 20-30 μL of testing volume and antibody volume, which provides venture for new highly sensitive small volume setup for HTS. This could be performed either manually or by using specialized robotic liquid handling systems. Selected glucocorticosteroids for secondary screening are often used for their immunosuppressant and anti-inflammatory activity which brings their mode of action by inhibiting classical NFkB-p65 pathway (Yasir et al., 2020). This further would have inhibited CINC-3 production as its production is controlled by key gene regulator NF-κB (Takaishi et al., 2000). Additionally, upregulation in the GFAP expression which is considered an hallmark for reactive astrocytes (Liddelow and Barres, 2017). However, when we assessed the changes in GFAP protein levels in cytokine combination treatment and glucocorticosteroid-treated groups, we did not see any change. This outcome was comparable with the reported data which showed that at *in vitro* level upon cytokine (TNF-α and or IL-1β) treatment, the GFAP expression was reduced instead of increased in *in vitro* models (Hyvärinen et al., 2019).

In conclusion, treatment with cytokine combinations successfully induced acute and chronic-like inflammatory conditions in MGC associated with SCI at the preclinical level. This model addresses limitations of current *in vitro* models of SCI. Pro-inflammatory cytokine combination treatments showed that different cytokines influenced the different level of expression of neuroinflammatory pathways. From the acute to the chronic phases, NF-κB and MAPK gene regulators showed variable effects on the modulation of pro-inflammatory milieu. One can use this approach to investigate future treatments and conduct screenings to evaluate drug or molecule's efficacy before proceeding for preclinical studies. Importantly, this platform can be used to screen therapeutic interventions for neuroinflammatory diseases, including SCI using high-throughput platforms.

### Limitations of the study

One of the limitations of the study is that, as several factors influence the glial cells, further studies would be needed to encompass the entire complexity of SCI. For example, future models should take into consideration the response of myelin debris or blood-born immune cells for reliable drug testing for the respective mode of action.

### Resource availability

#### Lead contact

Further information and requests for resources should be directed to and will be fulfilled by the lead contact, Abhay Pandit (abhay.pandit@nuigalway.ie).

#### Material availability

This study did not generate new unique reagents.

#### Data and code availability

All data supporting the results can be found in this manuscript and as Supplemental information. Data requests can be addressed to the corresponding author.

## Ethics statement

In this study, we used female Sprague-Dawley rats (Charles River UK Ltd., Margate, UK). All housing and procedures carried out in this study were approved by the Animal Care Research Ethics Committee (ACREC) at the National University of Ireland, Galway.

## Methods

All methods can be found in the accompanying Transparent methods supplemental file.

## Supplemental information

Document S1. Transparent methods, Figures S1–S11, and Tables S1 and S2

Data S1. Normalised data from proteome profiler array for IPA analysis, related to Figure 4

Data S2. Upstream regulators, related to Figures 4 and S3

Data S3. Disease and functions upon cytokine combination and LPS treatment, related to Figure 5

Data S4. Rates of ATP production from glycolysis and oxidative phosphorylation, related to Figure 6

## Footnotes

Supplemental information can be found online at https://doi.org/10.1016/j.isci.2021.102182.
