# Supplementary material for: A robust platform for high-throughput screening of therapeutic strategies for acute and chronic spinal cord injury
Source: iScience. 2021 Feb 12;24(3):102182. doi: 10.1016/j.isci.2021.102182 (PMC7921603; doi:10.1016/j.isci.2021.102182)
Supplement: Document S1. Transparent methods, Figures S1–S11, and Tables S1 and S2 [file mmc1.pdf]

iScience, Volume *24*

Supplemental information

A robust platform for high-throughput screening of therapeutic strategies for acute and chronic spinal cord injury

Vaibhav Patil, Enda O'Connell, Leo R. Quinlan, Howard Fearnhead, Siobhan McMahon, and Abhay Pandit

Supplemental Information

Table S1: Groups of cytokine combination treatments for 21 days study. Related to Figure 3.

Ctrl: Control, M-Ctrl: Media control. All used with 10 ng/mL of dose.

	TNF-α	IL-1β	IL-6	LPS	Time (Days)
1	+	-	-	-	1-21
2	-	+	-	-	-//-
3	-	-	+	-	-//-
4	+	+	-	-	-//-
5	-	+	+	-	-//-
6	+	-	+	-	-//-
7	+	+	+	-	-//-
8	-	-	-	+	-//-
9	Ctrl	Ctrl	Ctrl	-	-//-
10	M-Ctrl	M-Ctrl	M-Ctrl	-	-//-

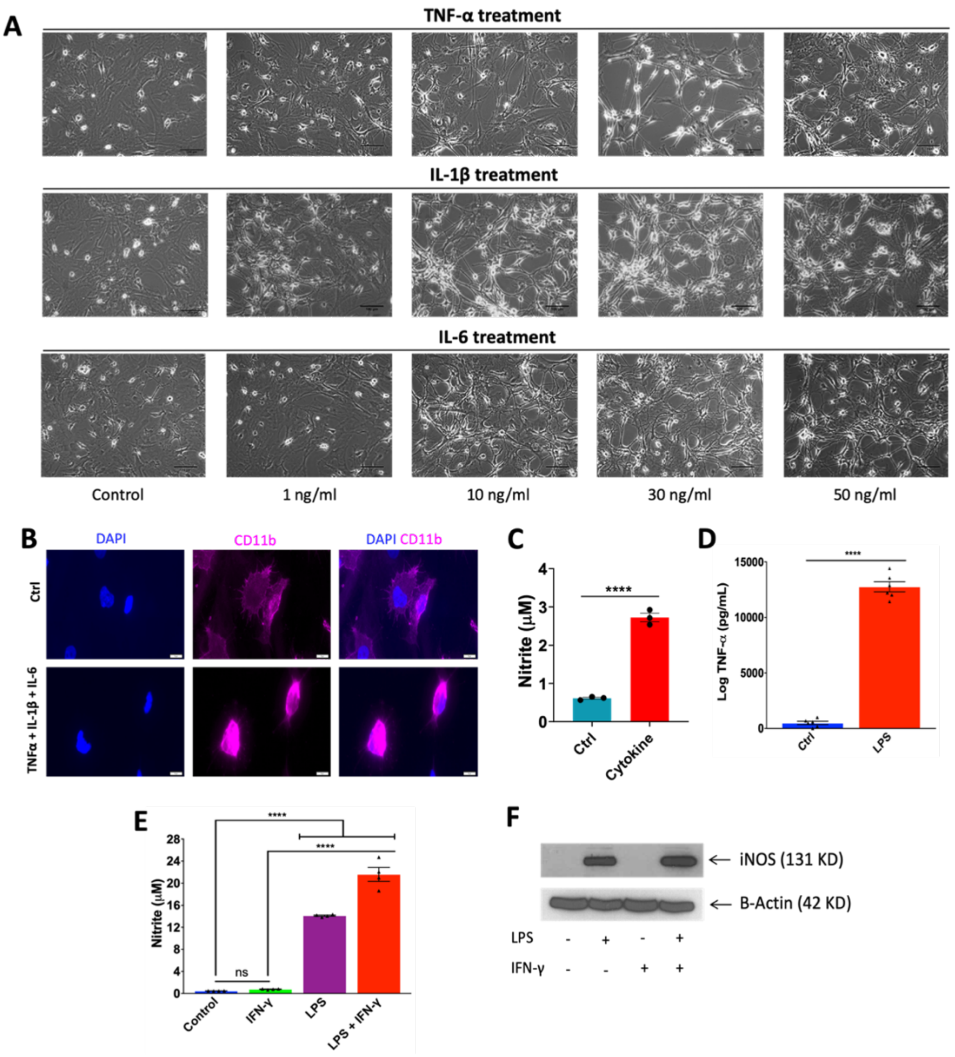

Figure S1: The cytokine combination treatment induces morphological changes in MGC. Related to Figure 2.

(A) Individual treatment of TNF-α, IL-1β and IL-6 with the concentration of 1, 10, 30 and 50 ng/mL showed morphological changes in MGC. Scale bar=100 µm. (B) Morphological changes in microglia as they became more ameboid shape upon cytokine combination treatment. Scale bar= 10 µm. (C) Griess assay showing the effect of cytokine combination (cytokine) on nitrite production. Data are expressed as mean ± SEM, n=three independent experiments with two technical replicates; $^{****}p<0.0001$, student *t*-test. (D) TNF-α expression after lipopolysaccharide (LPS) treatment. Data are expressed as mean ± SEM, n=three independent experiments with two technical replicates; $^{****}p<0.0001$, student *t*-test. (E) Griess assay showing the effect of LPS (100 ng/mL) and IFN-γ (10 ng/mL) on nitrite production. Data is expressed as mean ± SEM, n= two independent experiments with four technical replicates; $^{***}p<0.001$, $^{*}p<0.05$, one-way analysis of variance (ANOVA), post hoc Tukey test. (F) Western blot showing expression of iNOS upon treatment of LPS and LPS + interferon (IFN)-γ. Based upon these results, 100 ng/mL dose of LPS was selected for further studies.

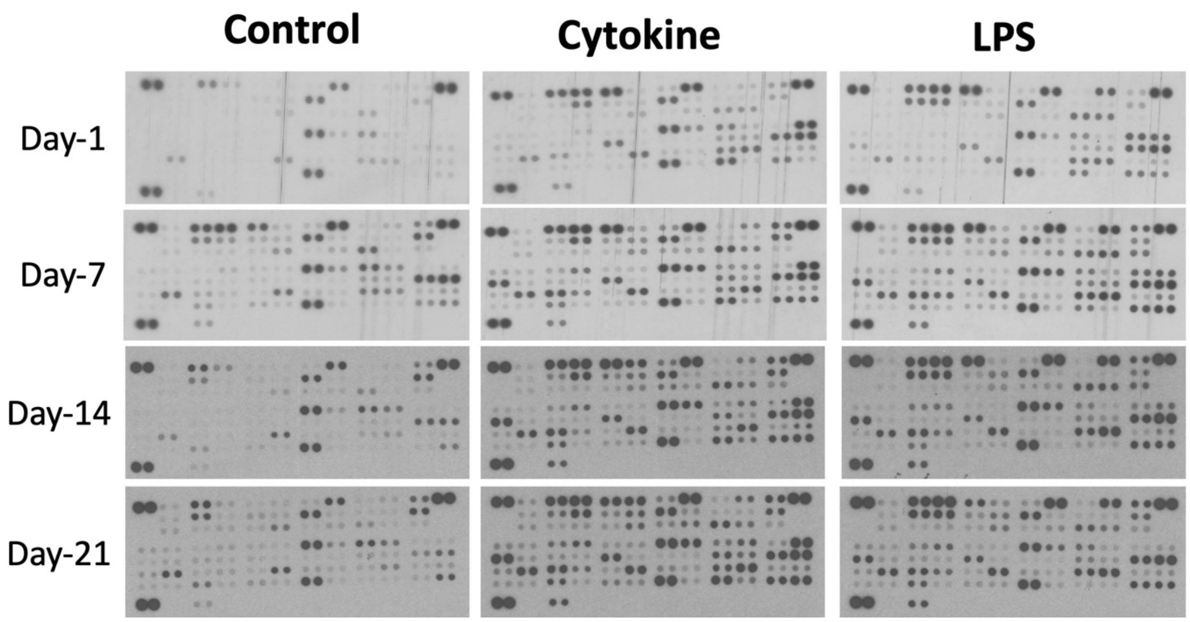

Figure S2: The Rat XL Cytokine Array detects multiple cytokines, chemokines, growth factors and other soluble proteins in the media (supernatant). Related to Figure 4.

Twelve blots for four time points (day-1, day-7, day-14 and day-21) and three conditions (Control, cytokine combination (10 ng/mL of each cytokine), and LPS (100 ng/mL)). Three experimental replicates pulled supernatant (media) run for two technical replicates (as each analyte was in duplicate).

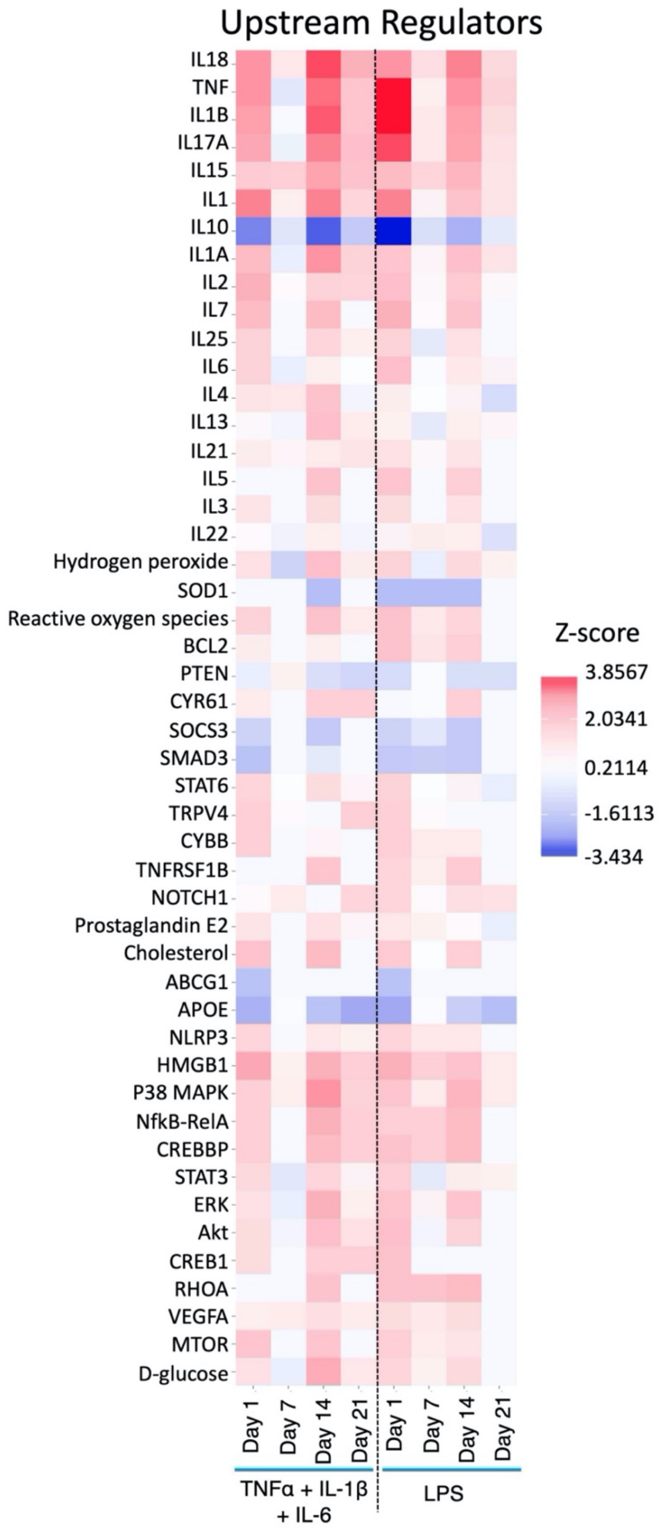

Figure S3: Differential regulation of upstream regulators upon cytokine and LPS induction. Related to Figure 4 and see also Data S2.

The mean pixel density data obtained from the proteome profile™ array was normalised to control and analysed in Ingenuity Pathway Analysis (IPA)© software with the cut-off of 1.5 for downstream and upstream regulators. The data are represented as activation Z-score.

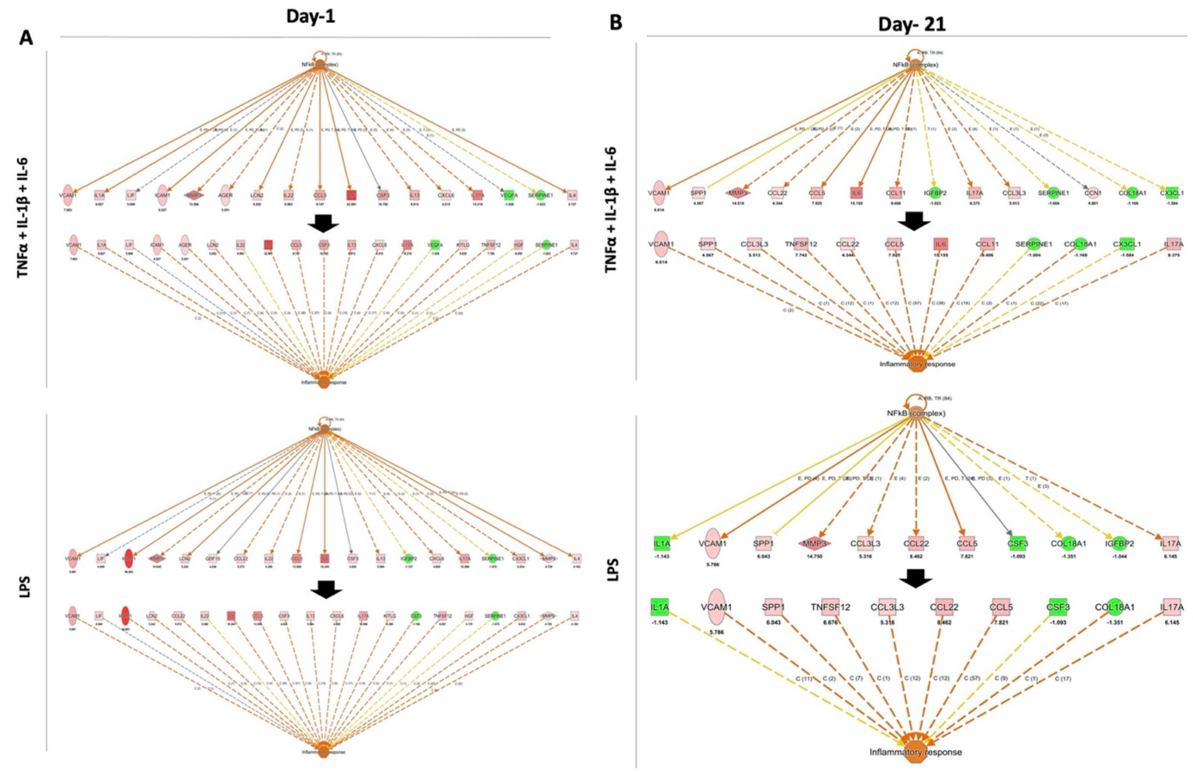

Figure S4: NFκB complex is directly or indirectly regulating the inflammatory response. Related to Figure 4.

Proteome profiler dataset was analyzed using IPA to demonstrate the prediction of upstream genes in NFκB complex and respective inflammatory response for each time point. The prediction shows a correlation between genes involved in NFκB pathway activation and inflammatory response upon cytokine combination (TNFα + IL-1β + IL-6) and LPS treatment at (A) day one and (B) day 21. The level of activation and type of genes involved were differentially regulated from day one to day 21 in both cytokine combination and LPS treatments. Also, between these two treatments, the level of activation and type of genes involved were unique. According to IPA, straight arrow: direct effect, dotted arrow: indirect effect and lines show predicted inhibition (green) or activation (orange) of the downstream genes.

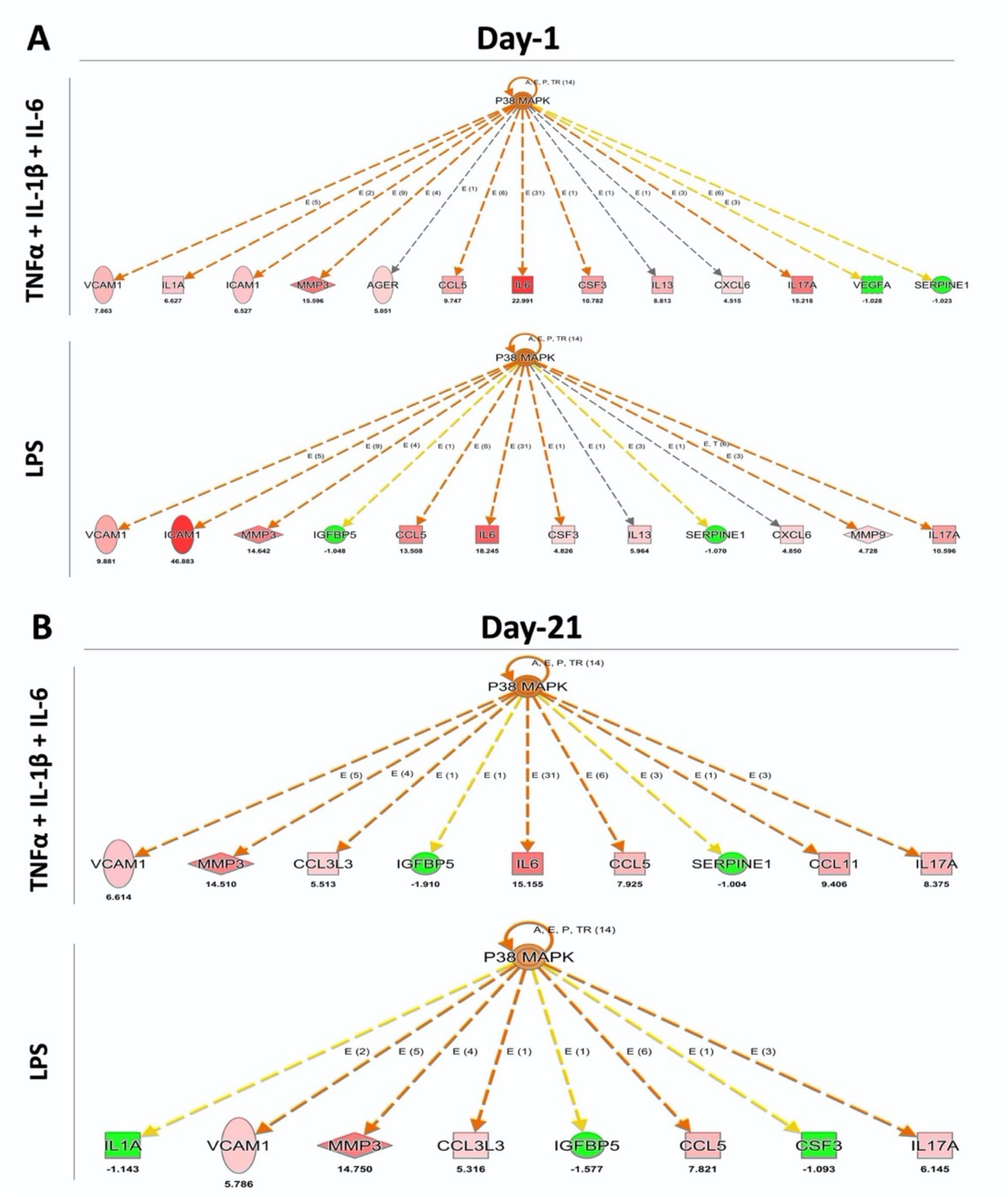

Figure S5: The p38-MAPK indirectly regulating genes involved in the inflammatory response. Related to Figure 4.

Proteome profiler dataset was analysed using IPA to demonstrate the prediction of upstream genes in the p38-MAPK pathway for each time point. The prediction shows a correlation between genes involved in the MAPK-p38 pathway leading to inflammation upon cytokine combination (TNFα + IL-1β + IL-6) and LPS treatment at (A) day one and (B) day 21. The level of activation and type of genes involved were differentially regulated from day one to day 21 in both cytokine combination and LPS treatments. Also, between these two treatments, the level of activation and type of genes involved were unique. According to IPA, dotted arrow: indirect effect and lines show predicted inhibition (green) or activation (orange) of the downstream genes.

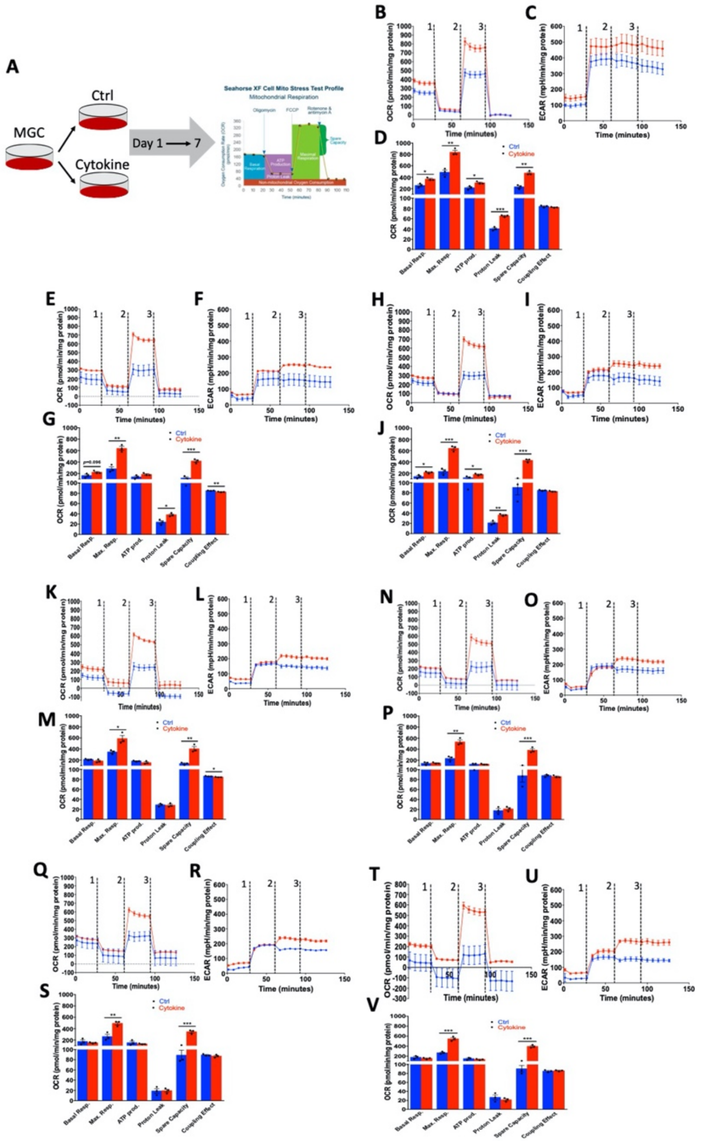

Figure S6: A cytokine combination treatment increases oxygen consumption rate (OCR), respiration, ATP production, proton leak and decreases coupling efficiency over seven days. Related to Figure 6. All parameters after performing seahorse XF cell mito stress test were calculated as a function of a cytokine combination treatment. For this, total protein per well was calculated using bicinchoninic acid (BCA) protein quantification assay and data was normalised against it. (A) Experimental plan. Day one (B-D), Day two (E-G), Day three (H-J), Day four (K-M), Day five (N-P), Day six (Q-S) and Day seven (T-V). (B, E, H, K, N, Q and T) OCR after the addition of three drugs (i.e. oligomycin, FCCP and Rotenone and antimycin A) sequentially. (C, F, I, L, O, R and U) Extracellular acidification rate (ECAR) after the addition of the above mentioned three drugs sequentially. (D, G, J, M, P, S and V) Six parameters: basal respiration, maximal respiration, ATP production, proton leak, spare capacity and coupling efficiency were measured and plotted as a bar graph. Data are represented as mean ± SEM, n= three experimental replicates. *p<0.05, **p<0.01, ***p<0.001 vs respective day of control. Unpaired t-test was performed to test the difference between the treatment and the respective day of control.

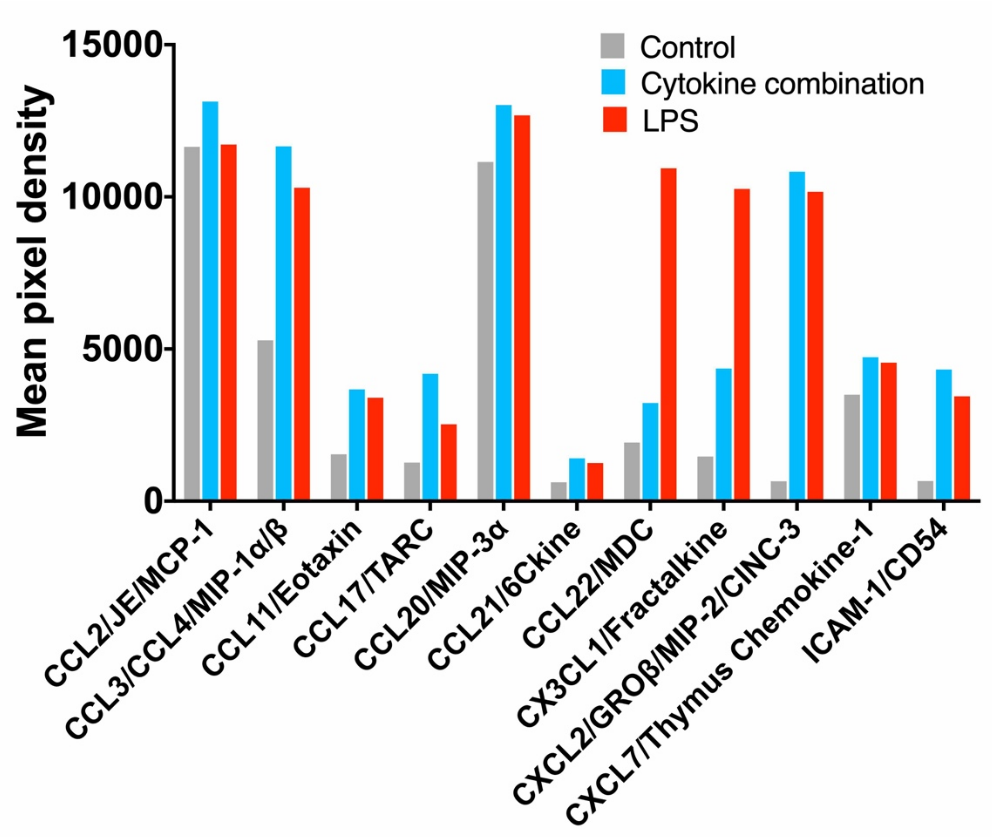

Figure S7: A panel of chemokines secreted by MGC after 24 hrs of treatment in the supernatant. Related to Figure 7.

The proteome profiler array data was analysed, and the mean pixel density plotted. A cytokine combination (i.e. TNF-α, IL-1β and IL-6 combination) was used along with LPS as a positive control. Each analyte was in duplicate. Based upon this data, cytokine-induced neutrophil chemoattractant (CINC)-3 (also called as CXCL2/ GROβ or MIP-2) was used for high-throughput screening (HTS) assay development.

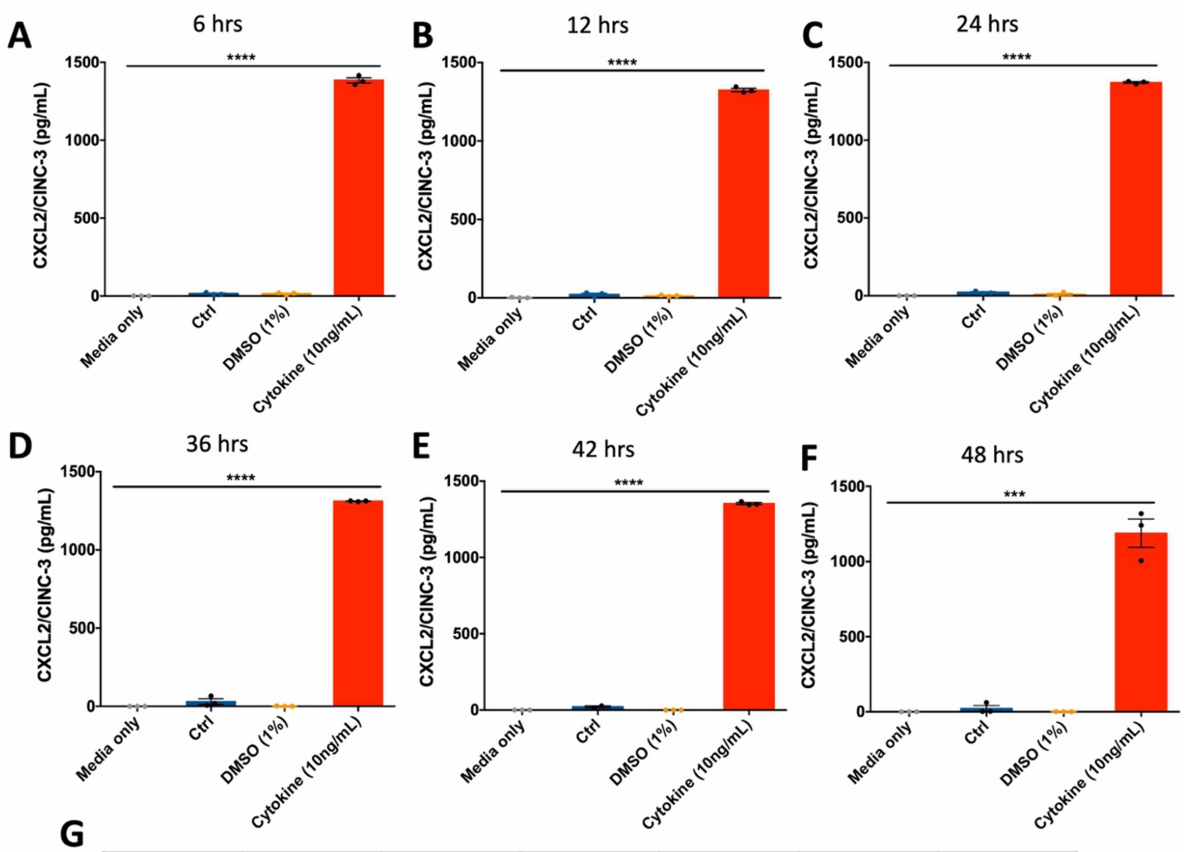

G

Hrs	6	12	24	36	42	48
Z' factor	0.92	0.93	0.96	0.92	0.95	0.49

Figure S8: CXCL2/CINC-3 expression after cytokine combination treatment. Related to Figure 7. (A-F) CXCL2/CINC-3 production after the cytokine treatment after 6 (A), 12 (B), 24 (C), 36 (D), 42 (E) and 48 (F) hours. Data are represented as mean ± SEM, n= three experimental replicates, $^{****}p<0.0001$ and $^{***}p<0.001$; one-way ANOVA followed by *post hoc* Tukey test. (G) quality assessment of the assay: Z' factor after 24 hrs of cytokine treatment was higher than at other time points.

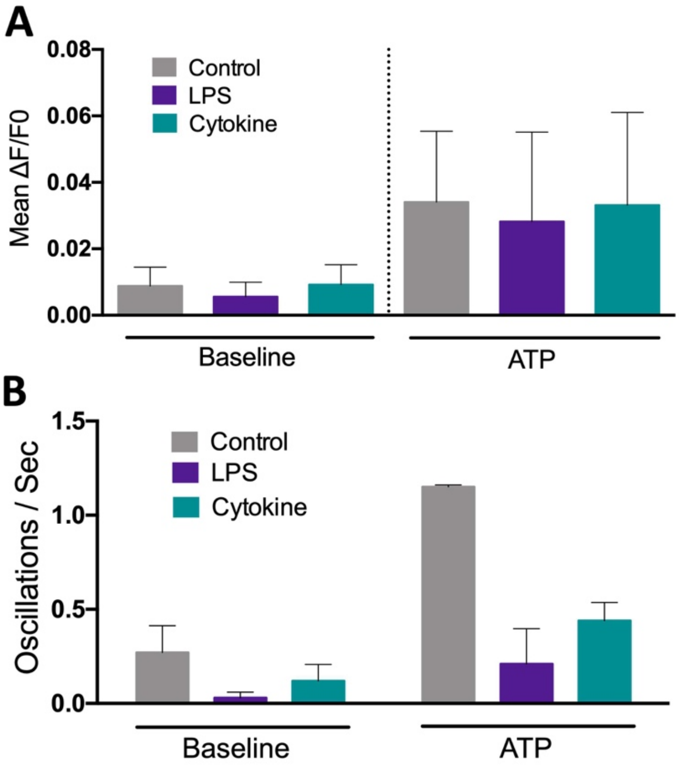

Figure S9: Effect cytokine combination and LPS on ATP induced endogenous intracellular Ca^{2+} signalling. Related to Figure 6.

MGC exhibited no effect of treatments on (A) amplitude (ΔF/F) of Ca2+ transient. However its (B) frequency was decreased upon both the treatments. Data is represented as mean ± SD, n= three technical replicates.

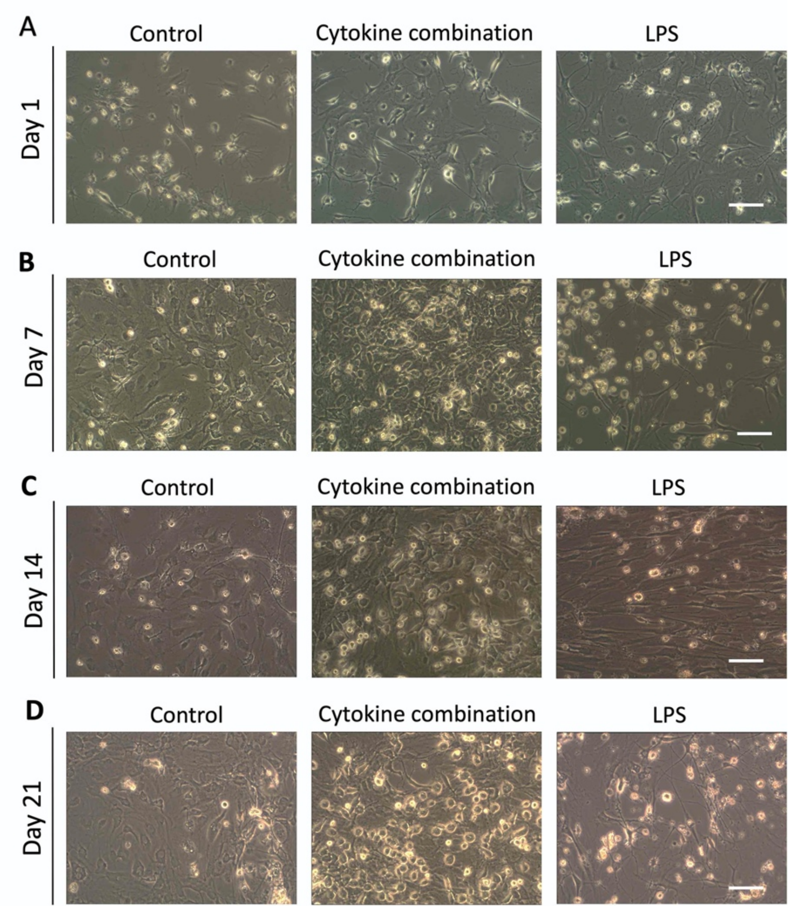

Figure S10: Phase contrast images of mixed glial cultures treated with cytokine combination and LPS at (A) day one, (B) day seven, (C) day fourteen and (D) day 21. Scale bar= 100 µm. Related to Figure 2.

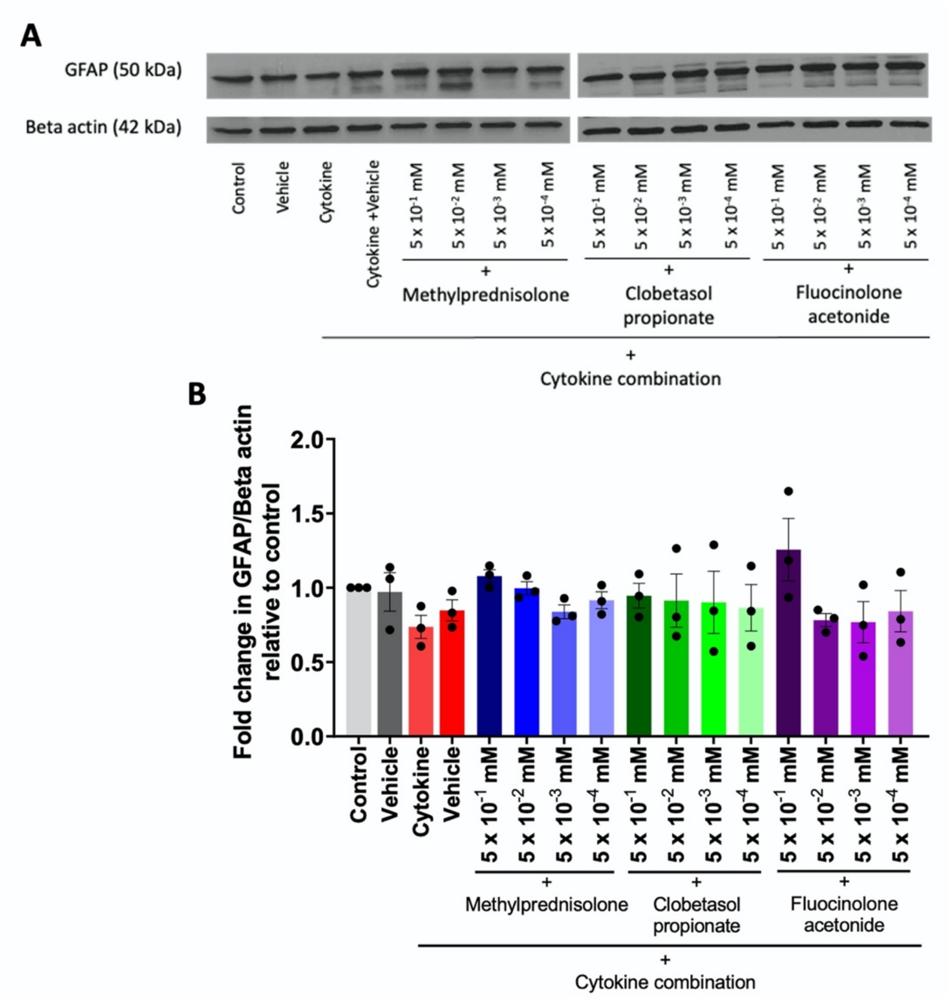

Figure S11: Validation of HTS- GFAP expression upon cytokine combination and corticosteroids treatment during secondary screening. Related to Figure 8.

(A) Western blots showing the expression of GFAP. We have selected three drugs from our primary screening, Methylprednisolone, Fluocinolone acetonide and Clobetasol propionate were treated with a concentration of 5 x 10-1 mM to 5 x 10-4 along with a cytokine combination treatment for 24 hrs. (B) Quantitative analysis of GFAP expression normalised with beta actin. Data are represented as mean ± SEM, n= three experimental replicates. One Way ANOVA. The fold change in GFAP expression was unchanged.

Table S2: Primary targets identified after high throughput screening. Related to Figure 7 and 8.

	(%) Alamar Blue reduced	CINC-3 (pg/mL)	Drug name	Function/ Use
1	96.52	551.53	Clobetasol Propionate	Corticosteroid, anti-inflammatory, antipruritic and vasoconstrictive
2	30.74	314.91	Idarubicin HCl	Cytotoxic anthracycline
3	110.30	534.04	Fluocinolone Acetonide	Anti-inflammatory and corticosteroid
4	105.62	699.12	Zidovudine (3'-Azido-3'-Deoxythymidine)	Thymidine analogue, reverse transcriptase inhibitor and inhibits protein glycosylation
5	104.18	790.36	Methylprednisolone	Anti-inflammatory and corticosteroid
6	7.67	160.32	Auranofin	Cytotoxic
7	110.26	-286.59	Goserelin Acetate	GnRH agonist
8	18.60	-2.03	Daunorubicin HCl	Cytotoxic anthracycline
09	42.14	428.22	Doxorubicin HCl	Cytotoxic anthracycline
10	103.48	792.78	Cefotaxime Acid	Semisynthetic cephalosporin antibiotic with bactericidal activity
11	117.78	456.27	Nystatin	Polyene antifungal drug
12	19.39	-81.41	Dactinomycin (= Actinomycin D)	Cytotoxic
13	32.59	356.48	Epirubicin HCl	Cytotoxic anthracycline

Transparent Methods

Cell culture

Primary MGCs were prepared from spinal cords as previously described (Kilcoyne et al., 2019). Spinal cords were isolated by the hydraulic extrusion technique from three-day-old postnatal rats, with minor modification in the spinal cord extrusion method (Kennedy et al., 2013). MGCs were cultured as previously described with slight modification in protocol (McCarthy and de Vellis, 1980). Briefly, meninges were gently peeled from spinal cords under a microdissection microscope. Spinal cords were chopped into fine (approx. 1 mm) pieces and digested using 1% trypsin-EDTA solution for 15-20 minutes. Trypsin activity was inhibited using DMEM-high glucose medium supplemented with 10 % fetal bovine serum (FBS) and 1% penicillin/streptomycin. Tissue was further digested by triturating it through various sizes of needles starting from 19G, 18G and then 23G. The digested tissue was expelled through a cell strainer filter (70 μM, Nitex Mesh, Falcon™), centrifuged and seeded into respective flasks. The MGCs were grown *in vitro* for three weeks before treatments were applied. Passage number one (P1) was maintained throughout all experiments.

Immunocytochemistry, microscopy and image analysis

MGC cells with a density of 1 X 10^5 cells/well were grown for two days on poly-L-lysine (PLL) coated coverslips in 24-well plates. On the day of staining, media was removed, and cells were washed with phosphate buffer saline (PBS). Fixed cells were permeabilized by incubating them in 0.2% Triton X-100 in PBS, followed by washing with PBS. Non-specific binding was blocked with 1% bovine serum albumin (BSA)+ 10% normal goat serum (NGS) in PBS solution (blocking buffer) for one hr at room temperature (RT). Primary antibody rabbit anti-GFAP (1:500, Dako, Z033429), mouse anti-CD11b (1:200 Sigma, CBL1512), mouse anti-Tubulin beta III isoform (1:200, Millipore, MAB1637) and rabbit anti-Olig2 (1:200, Millipore, AB9610) were prepared in blocking buffer and added to the plates, which were incubated overnight at 4°C. After washing, cells were subsequently incubated with secondary antibody Alexa Fluor® 488 (1:500, Thermo Scientific™, A-10667) and Alexa Fluor® 546 (1:500, Thermo Scientific™, A11035) for one hr at RT. After washing, cells were then incubated in Hoechst 33342 (1:2000, Thermo Scientific™, 62249) for five min at RT. Coverslips were mounted onto glass slides using fluoromount and observed under the inverted fluorescent microscope.

Fluorescent cytochemistry images were captured on an Olympus VS120 Virtual Slide Microscope with Olympus VS fluorescence software (VS-ASW-FL). Every image for each time point was taken at the same exposure so that fluorescence intensity was consistent. Additionally, fluorescence intensity (FI) of lectin staining was quantitatively analysed using the ImageJ (Fiji) software. The number of Hoechst-

positive nuclei was quantified. Astrocytes cell morphology was assessed by two parameters, length and roundness of astrocyte processes, which were further, calculated by NeuronJ plugin and roundness measurement, respectively.

Flow cytometry:

For flow cytometry, mixed glial culture (MGC) cells were washed, trypsinised and suspended in flow cytometry staining (FACS) buffer. Cells were strained (Cell Trix®, 50 µm) and fixed with 2% paraformaldehyde (PFA) for 15 min at room temperature (RT) and washed with FACS buffer. Cells were permeabilised with 0.3% Triton in phosphate buffer saline (PBS) for 20 min at RT (not for the CD11b surface marker) and incubated in 3% BSA + 10% normal goat serum (NGS) for 30 min at RT. Cells were incubated with anti-glial fibrillary acidic protein (GFAP)- Alexa Fluor® 647 (1:20, BD Biosciences, 561470) and anti-CD11b/c-PE (1:20, ImmunoTools, 23159114) made in 1% BSA/PBS solution, for one hr at RT on ice. Cells were then washed with 1% bovine serum albumin/phosphate buffer saline (BSA/PBS), centrifuged, re-suspended in FACS buffer and acquired in the flow cytometer.

Optimisation of concentration of cytokines

MGCs were seeded at $5x10^5$ cells/well on poly-L-lysine (PLL) coated six-well plates or in 24-well plates. After growing in an incubator for two days, the media was replaced with no-serum media (Dulbecco's modified Eagle's medium (DMEM + 1% Penicillin/Streptomycin (P/S)). Cells were treated with four cytokine concentrations: 1, 10, 30 and 50 ng/mL, for 24 hrs.

Measurement of nitrite production

The nitrite concentration was assessed by the colorimetric reaction of the Griess reagent (Molecular Probes, Inc., G-7921). Briefly, after treatments on MGC for specific time points, the supernatant was collected from each group, and an assay was performed according to the manufacture's protocol. Griess reagent prepared nitrite concentration was calculated from a standard curve using sodium nitrite.

Study design for chronic inflammation (21 days) of MGC

MGCs were seeded at $5x10^5$ cells/well on PLL-coated six-well plates or in 24-well plates. After growing in an incubator for two days, media was replaced with low-serum media (DMEM + 1% P/S + 1% FBS) and cells were treated with 10ng/mL of three cytokines (TNF-α, IL-1β, and IL-6) (R & D Systems; recombinant rat TNF-alpha protein, 510-RT-010; recombinant rat IL-1 beta/ IL-1F2 protein, 501-RL-010; recombinant rat IL-6 protein, 506-RL-010) at day zero and every subsequent two days (i.e. day 0,

2, 4, 6, 8, 10, 12, 14, 16, 18 and 20) until day 21. Multiple combinations of these three cytokines were used as listed in Table S1. At each of the treatment time points (except day zero), half of the media was changed and filled with new cytokine treatment media. 100ng/mL dose of LPS (Sigma-Aldrich) was given in the same procedure as a positive control. Finally, a negative control was carried out by performing the same half-media change procedure with low-serum media. All experiments were run with three biological replicates. The supernatant was collected, and protein was isolated at respective time points for further analysis.

Western blotting

MGC cells of density 5 x 10^5 cells/mL were seeded into six-well plates and after respective treatments, cells were lysed in a radioimmunoprecipitation assay (RIPA) buffer (50 mM Tris-HCl, pH 8.0, 150 mM NaCl, 0.02% sodium azide, 0.1% sodium dodecyl sulphate (SDS), 1% Nonidet P-40, 0.5% sodium deoxycholate) (Sigma-Aldrich, R0278) with a protease inhibitor cocktail (1:100) (cOmplete™, EDTA-free, Roche, Inc., 11873580001), phenylmethylsulfonylfluoride (1:50) (Sigma-Aldrich, 93482) and phosphatase inhibitor cocktail (1:10) (PhosSTOP™, Roche, Inc., 04906845001). The protein concentration in the total cell lysate was determined using a bicinchoninic acid (BCA) protein assay kit (Pierce™ BCA Protein Assay Kit, Thermo Fischer Scientific, 23225). An equal amount of protein from each sample was separated by 10-12% SDS polyacrylamide gel electrophoresis and transferred to Hybond® ECL™ nitrocellulose membrane. Membranes were stained with Ponceau S to visualize successful transfer and protein loading. The membrane was blocked with either 5% milk or 5% BSA depending upon the suitability of primary antibodies to avoid non-specific binding of antibodies. This was followed by primary antibody incubation overnight at 4°C with 1:2000 rabbit anti-P-NFκB-p65 (Ser536) (93H1) (Cell Signalling, 3033s), 1:200 mouse anti-NFκB-p65(F-6) (Santacruz Biotech, sc-8008), 1:1000 P-p38 MAPK (Thr180/Tyr182) (Cell Signalling, 3033s), 1:200 mouse anti-p38α/β MAPK (Santacruz Biotech, sc-7972), 1:1000 mouse anti-GFAP (Sigma, G3893), 1:2000 rabbit iNOS (Thermo Fisher Scientific, PA3-030A) or one hour at RT with 1:15,000 anti-β-actin (Sigma, A5441) on a rocking platform. All washing steps were carried out in Tween-20 in TBS (0.1%). Next, horseradish peroxidase-conjugated secondary goat anti- rabbit or goat anti-mouse antibodies (prepared in 5% milk or 5% BSA at 1:10,000) were applied, followed by enhanced chemiluminescence detection. Signals from protein bands were captured on x-ray films (CL-XPosure™ Film, Thermo Scientific™, 34090), which were further analysed using Image Studio™ Lite software and signals recorded as pixel density.

Proteome profile array

The Rat XL Cytokine Proteome Profiler™ Array kit (ARY030, R&D SYSTEMS) was used to assess the parallel determination of relative expression of chemokines, pro- and anti-inflammatory cytokines and growth factors secreted by MGCs. The assay was carried out as per the manufacturer's procedure given for the supernatant. Positive signals could be seen on X-ray films (CL-XPosure™ Film, Thermo Scientific™, 34090) which were further analysed using Image Studio™ Lite software and signals recorded as pixel density. Further 'mean pixel density' from each analyte was calculated, and the data plotted as a heatmap. Hierarchical clustering of the data was performed using Morpheus, https://software.broadinstitute.org/morpheus.

Ingenuity pathway analysis (IPA)

The proteome profiler™ data were normalised with the control (untreated) group and uploaded into IPA software© (QIAGEN). Based upon distribution analysis of the data, a cut-off of 1.5 for downstream molecules and upstream molecules was set. A comparative analysis was carried out to understand the effect of molecules on biological functions and diseases. Further regulator network analysis and canonical pathway analysis were performed.

Cellular stress assay

40,000 MGC cells/well were seeded into the XFp Seahorse miniplates. After growing in an incubator for two days, the cytokine treatment was given only for seven days as described above in the "Study design for chronic inflammation of MGC" section. On the day of assay, the media was replaced with glucose, pyruvate, L-glutamine and phenol red-free Seahorse XF base medium. Assay medium was prepared with the addition of glucose (25 mM), sodium pyruvate (1.0 mM) and L-glutamine (2.0 mM). The pH was adjusted to 7.4 using 1N NaOH, and the media was filtered through a 0.2 mm filter. Before the assay, the sensor cartridge was hydrated overnight, and Seahorse instrument was turned on at least five hrs before the assay. Oxygen consumption rate (OCR) and extracellular acidification rate (ECAR) was measured on an XFp Seahorse analyser with the sequential addition of one. Oligomycin (1 uM), 2. FCCP (2 uM), 3. Rotenone/antimycin (0.5 uM) for 110 minutes with five readings per cycle.

After the assay was completed, cells were lysed in RIPA buffer, and the protein concentration was determined using a BCA protein assay kit (Pierce™ BCA Protein Assay Kit, Thermo Scientific™, 23225). OCR and ECAR data were normalised on Wave Controller software using protein concentration per well. The extracellular acidification and rate of adenosine triphosphate (ATP) production by glycolysis and oxidation were calculated using ECAR and OCR values (Mookerjee et al., 2015).

In vitro reactive oxygen species *(*ROS)/superoxide detection

20,000 MGC cells/well were seeded into the specialised PLL coated 384-well plates (Greiner Bio-one CELLCOAT®). After growing for two days, the media was replaced with FBS-free media (DMEM +1% P/S). Cells were treated with a combination of pro-inflammatory cytokines (tumour necrosis factor-α (TNF-α), interleukin (IL)-1β and interleukin (IL)-6) of dose 10 ng/mL for 24 hrs. After the treatment, media was removed, and the protocol was followed as given in ROS-ID® total ROS/superoxide detection kit, ENZ-51010. Fluorescence was measured at Ex/Em: 490/525 nm to detect ROS in cells and data was presented as a bar graph.

Calcium imaging

Calcium imaging was performed as previously described with some modifications (Avazzadeh et al., 2019). After 24 hr of treatments, MGCs were washed with warm Krebs-Ringer's buffer, incubated with 1µM Fluo-4 AM (ThermoFisher scientific, F14201) in Krebs-Ringer's buffer for 40 min at 37°C in the incubator. Cells were further washed and left with fresh Krebs-Ringer's buffer for 20 min in the incubator. Images were taken in an imaging chamber (Warner Instruments, Inc., RC-26GLP) on a Zeiss Axiovert 200 microscope (× 10) for total 180 sec, 60 sec with baseline and 120 sec with adenosine triphosphate (ATP) (10 µM) as an inducer. Videos were captured with a Hama-matsu ORCA284 at 1 Hz frame rate. FluoroSNNAP in MATLAB (MathWorks, Inc.) was used to analyse images. A fluorescence ratio ($\Delta F/F_0$) and oscillations per sec were calculated.

Screening assay development (optimisation and validation of ELISA assay to detect CINC-3)

15,000 MGC cells/well were seeded into the specialized PLL-coated 384 well plates (Greiner Bio-one CELLCOAT®). After growing for two days, the media was replaced with 50 µL of FBS-free media (DMEM +1% P/S) and cells were treated with a combination of pro-inflammatory cytokines (TNF-α, IL-1β, and IL-6) of dose 10 ng/mL (per cytokine) for 6, 12, 24, 36, 42 and 48 hr. After the treatment, 30 µL of media was collected, and ELISA (CINC-3) (DuoSet®, Rat CXCL2/CINC-3 ELISA, DY525, R&D Systems) was performed using high binding affinity 384-well clear plates (Nunc® MaxiSorp™ 384-well plates, Sigma, P6366). Assay Z' factor was calculated to evaluate the suitability of this assay (Zhang et al., 1999).

$$\text{Z}'\text{Factor} = 1 - \frac{3(\sigma_\rho + \sigma_n)}{|\mu_\rho - \mu_n|}$$

This assay was performed using JANUS® automated liquid handling workstation (Perkin Elmer) and Thermo Multidrop 384 Dispenser (Thermo Fischer Scientific). 15,000 cells/well were seeded into the specialized PLL-coated 384 well plates. After the treatment, 30 µL media was transferred to high

binding affinity 384-well clear plates using a JANUS® workstation to perform ELISA (CINC-3). Later steps were carried out using a Thermo Multidrop 384 Dispenser. Further, the Z' factor was calculated to evaluate the suitability of this assay under automated conditions.

High-throughput drug screening to identify hits with anti-inflammatory properties

15,000 MGC cells/well were seeded into the specialized PLL-coated 384 well plates (Greiner Bio-one CELLCOAT® using JANUS® automated workstation, and the treatment was given after two days. On the day of treatment, the drug compounds (10 mM) were resuspended in 60 µL of FBS-free media (DMEM +1% P/S) with the combination of pro-inflammatory cytokines (TNF-α, IL-1β, and IL-6) of dose 10 ng/mL (per cytokine). Drugs were incubated for one hr at RT. Further, media from the cell containing PLL-coated plates was removed, and 50 µL of media/treatment containing drug compounds was transferred to cell plates. The final concentration of compounds was 3 µM and 0.03% DMSO v/v in each assay well. After 24 hr of treatment, 30 µL of supernatant from each assay well was transferred to high binding affinity 384-well clear plates to perform ELISA for CINC-3). Later steps were carried out using Thermo Multidrop 384 Dispenser. The optical density (450 and 540 nm) of each well was determined immediately, reading at 540 nm was subtracted from the reading at 450 nm. To analyse the effect of drugs on inflammation, a cut-off of 50% reduction in the CINC-3 expression was applied. Further to assess cell viability, 20 µL of 10% solution of alamarBlue™ was added per well containing 20 µL of previous treatment media. Fluorescence was measured at Ex 531/Em 595 after five hours of incubation in the incubator at 37°C. All samples were normalised to DMSO (0.03%) control group.

The secondary screening was performed using selected hit compounds such as methylprednisolone (LKT-M1877-M100, Enzo Life Sciences), fluocinolone acetonide (LKT-F4582-M025, Enzo Life Sciences) and clobetasol propionate (LKT-C4659-M100, Enzo Life Sciences) to validate the primary screening. 20,000 MGC cells/well were seeded into the specialized PLL-coated 96-well plates. After growing for two days, the media was replaced with 100 µL of FBS-free media (DMEM +1% P/S) and cells were treated with a combination of pro-inflammatory cytokines (TNF-α, IL-1β, and IL-6) at 10 ng/mL (per cytokine) for 24 hr. 30 µL of supernatant from each assay well was transferred in high binding affinity 384-well clear plates, and ELISA (CINC-3) was performed as per the manufacturer's protocol. Later alamarBlue™ assay (Bio-Rad) was performed on cells as per manufacturers protocol.

Statistical analysis

All statistical analyses were done using GraphPad Prism 8.00 software, Inc. Most data were analysed by one-way analysis of variance (ANOVA) followed by Tukey multiple comparison test for comparing more than three samples, and two-tailed unpaired *t*-tests for comparing two samples with 95% confidence. Non-parametric data were analysed by the Mann-Whitney U test for comparing two samples with 95% confidence. $p < 0.05$ was considered to be statistically significant.

Supplemental References:

Avazzadeh, S., McDonagh, K., Reilly, J., Wang, Y., Boomkamp, S. D., McInerney, V., Krawczyk, J., Fitzgerald, J., Feerick, N. & O'Sullivan, M. (2019). Increased ca 2+ signaling in nrxn1α+/− neurons derived from asd induced pluripotent stem cells. *Mol. Auti.,* 10**,** 1-16.

Kennedy, H. S., Jones, C., 3rd & Caplazi, P. (2013). Comparison of standard laminectomy with an optimized ejection method for the removal of spinal cords from rats and mice. *J. Histotech.,* 36**,** 86-91.

Kilcoyne, M., Patil, V., O'Grady, C., Bradley, C. & McMahon, S. S. (2019). Differential glycosylation expression in injured rat spinal cord treated with immunosuppressive drug cyclosporin-a. *ACS Omega,* 4**,** 3083-3097.

McCarthy, K. D. & de Vellis, J. (1980). Preparation of separate astroglial and oligodendroglial cell cultures from rat cerebral tissue. *J. Cell. Biol.,* 85**,** 890-902.

Mookerjee, S. A., Goncalves, R. L. S., Gerencser, A. A., Nicholls, D. G. & Brand, M. D. (2015). The contributions of respiration and glycolysis to extracellular acid production. *Biochim. Biophys. Acta.,* 1847**,** 171-181.

Zhang, J. H., Chung, T. D. & Oldenburg, K. R. (1999). A simple statistical parameter for use in evaluation and validation of high throughput screening assays. *J. Biomol. Screen.,* 4**,** 67-73.
